# Health equity in urban and rural settings: implementation of the place standard tool in Spain

**DOI:** 10.3389/fpubh.2024.1292032

**Published:** 2024-05-09

**Authors:** Ana Ocaña-Ortiz, Vicente Gea-Caballero, Raúl Juárez-Vela, Rosana Peiró, Elena Pérez-Sanz, Iván Santolalla-Arnedo, Teresa Sufrate-Sorzano, María Elena Garrote-Cámara, Joan Josep Paredes-Carbonell

**Affiliations:** ^1^Local Action and Health Equity Group (ALES Group), Foundation for the Promotion of Health and Biomedical Research in the Valencian Region (FISABIO), Valencia, Spain; ^2^Gandia Health Department, Gandia, Spain; ^3^Faculty of Health Sciences, VIU Valencia International University, Valencia, Spain; ^4^Research Group SALCOM Community Health and Care, Valencia International University, Valencia, Spain; ^5^Department of Nursing, University of La Rioja, Logroño, La Rioja, Spain; ^6^GRUPAC, University of La Rioja, Logroño, La Rioja, Spain; ^7^Publica Health General Directorate, Health Department, Generalitat Valenciana, Valencia, Spain; ^8^CIBERESP ISCIII, Madrid, Spain; ^9^Public Health Center of Alzira, Valencia, Spain

**Keywords:** social determinants of health, health equity, community participation, urban health, surveys and questionnaires, rural areas

## Abstract

The physical, social, and economic characteristics of neighborhoods and municipalities determine the health of their residents, shaping their behaviors and choices regarding health and well-being. Addressing local environmental inequalities requires an intersectoral, participatory, and equity-focused approach. Community participation plays a vital role by providing deeper insights into local contexts, integrating community knowledge and values into processes, and promoting healthier, fairer, and more equitable actions. In recent years, various tools have been developed to assess places and transform them into health-promoting settings. One such tool, the Place Standard Tool (PST), facilitates discussions on Social Determinants of Health grouped into 14 themes, serving as a starting point for local health interventions. In this study, that took place between August 2019 and February 2020, we described the resident’s perceptions of two municipalities in the Valencian Community, Spain, using the validated Spanish version of the PST. A mixed-method convergent-parallel design was used to gain a holistic insight into residents’ experiences concerning their physical, economic, and social environment. A total of 356 individuals from both municipalities participated in the study through discussion groups, structured interviews, and online survey. Descriptive analysis of the individual questionnaire answers was conducted, and differences between municipalities were explored. Qualitative thematic analysis was conducted on structured interviews and discussion groups. Quantitative and qualitative data were integrated to facilitate their comparison and identify areas of convergence or divergence in the findings. Overall, rural areas received more favorable evaluations compared to urban ones. Public Transport as well as Work and Local Economy were consistently rated the lowest across all groups and contexts, while Identity and Belonging received the highest ratings. In the urban area, additional negative ratings were observed for Traffic and Parking, Housing and Community, and Care and Maintenance. Conversely, Identity and Belonging, Natural Spaces, Streets and Spaces, Social Interaction, and Services emerged as the highest-rated themes overall. In the rural context, positive evaluations were given to Walking or Cycling, Traffic and Parking, Housing and Community, and Influence and Sense of Control. Significant differences (*p* < 0.01) between urban and rural settings were observed in dimensions related to mobility, spaces, housing, social interaction, and identity and belonging. Our study illustrated the capacity of the PST to identifying aspects within local settings that influence health, revealing both positive and challenging factors. Successful implementation requires appropriate territorial delineation, support from local authorities, and effective management of expectations. Furthermore, the tool facilitated community participation in decision-making about local environments, promoting equity by connecting institutional processes with citizen needs.

## 1 Introduction

The characteristics of neighborhoods, towns, and cities have the ability to influence the health and well-being of those who live in them, where the physical, social, and economic contexts that make them up are conditioned by the Social Determinants of Health (1). The unequal distribution of these factors exerts its impact along the axes of inequality (2), either enhancing or restricting the behaviors and choices of people living in these environments in relation to their health and well-being (3).

There is increasing evidence contributing to the understanding of how these structural factors operate through direct mechanisms, such as social interaction, physical activity, stress, safety, material deprivation, natural environment, and climate change (4–6). Furthermore, these mechanisms interrelate and can create synergies in their impact on health (5). In analyzing the effect of environmental characteristics on health, some studies examine their effect on healthy behaviors (3, 7–29), while others explore their relationship with chronic diseases and exposure to related risk factors (3, 15, 16, 30–37). Moreover, there is growing evidence about addressing the existing inequalities between urban and rural areas (5, 12, 22, 27, 38–43), where barriers to access to public transport, housing, basic services, and activities that promote social contact indicate the need to consider territory as a more explicit element in the analysis of the effects of environments on health.

Addressing social inequalities in the local environment poses a challenge, where acting on physical, social, and economic contexts requires an intersectoral, participatory, and equity-focused perspective (1). To influence these factors, the involvement of both policymakers and the community is necessary. Recent literature (44–48) emphasizes the importance of community engagement in the decision-making process regarding the design, planning, and implementation of policies and actions related to the transformation of neighborhoods and municipalities. Participation provides a deeper and more holistic view of places, integrates community knowledge and values into processes, builds consensus, and promotes empowerment and capacity-building spaces that foster healthier, fairer, and more equitable approaches (49).

In the integration of community participation in decision-making processes, multiple formats and different degrees of engagement have been adopted, ranging from individual consultations to collective community planning events (49). To assess places, multiple conceptual frameworks, guides, and participatory tools have been developed (44, 45, 50–54) with the aim of promoting community engagement in the transformation of their neighborhoods and municipalities.

The Place Standard Tool (PST) (55) has been developed in Scotland to assess places by promoting structured conversations around 14 topics related to the physical, social, and economic environment of a place. It is designed to facilitate an in-depth understanding of how a place affects people or groups involved in it. Furthermore, the PST enables the identification, characterization, and prioritization of action concerning the strengths and challenges pertaining to the impact of the place on health within a specified territory. This tool, included in the WHO compendium of tools (54) for developing healthy environments, has been implemented in different contexts and countries (56), and was recently translated, validated, and adapted into Spanish (57).

Therefore, in order to understand the performance of the Spanish adaptation of the PST in different settings in the Valencian Community, the objective of this study was to describe the community’s perception of their municipality in terms of physical, social, and economic aspects, and explore the differences between a rural and an urban context.

## 2 Materials and methods

### 2.1 Study design

**Figure 1:**
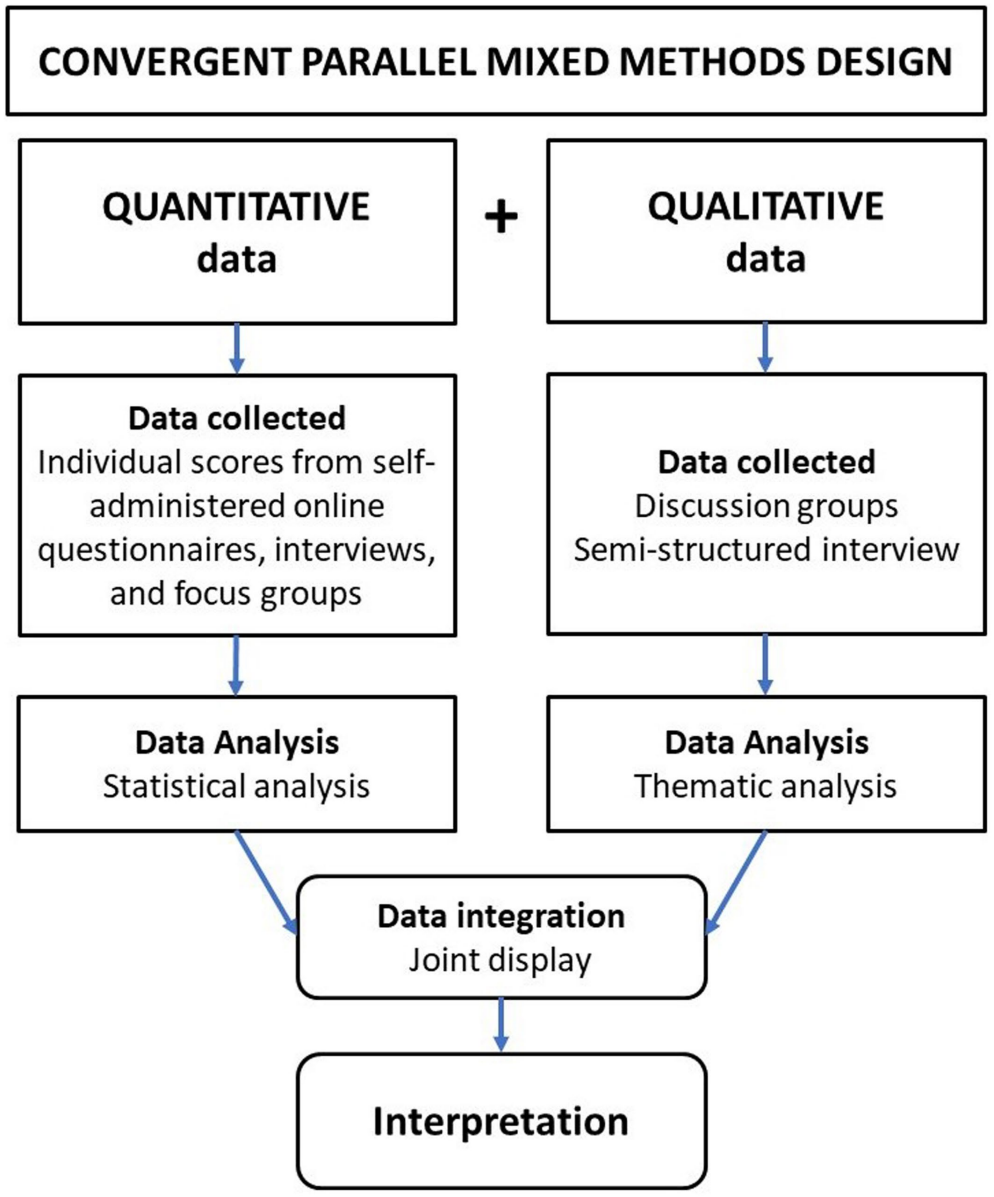
Schema for the mixed-method research design.

The study was conducted from August 2019 to February 2020 in two Spanish municipalities. We designed a convergent-parallel mixed-method study (QUAL+QUAN) (58). Qualitative and quantitative data were collected and analyzed separately, and their results were compared during the interpretation process and integrated into a joint display (Figure 1). Methodological and data triangulation were employed to integrate corroboration between qualitative and quantitative data to provide a deeper understanding of the impact of the physical, social, and economic environments in communities (59). The quality of the study was assessed using the Mixed Methods Appraisal Tool (MMAT) (60).

### 2.2 Study setting

The study was conducted in Denia (urban context) and Yátova (rural context), two municipalities of the Valencian Community, Spain, affiliated with the Network of Municipalities for Health of the Valencian Community, XarxaSalut (61). Denia is a coastal city in the province of Alicante with a population of 42,827 residents, of which 23.7% are of foreign origin (62). Its most important economic activity is residential tourism (63). The municipality of Denia consists of 10 neighborhoods and one outlying district. Yátova is a rural municipality located inland in the province of Valencia. It has a population of 2,079 residents, with over 25% of them being individuals aged 65 and older, and its primary economic activity is in the service sector (commerce, transportation, and hospitality) (64). Further details about the municipalities can be found in the Supplementary material (Supplementary Figure S1 and Supplementary Table S1).

### 2.3 Participant selection, procedure, and data collection

Participation was designed to ensure the representativeness of the population of the territory to be evaluated and the specific inclusion of groups organized according to sociodemographic characteristics of the municipality and the axes of inequality (2). To achieve this, three participation methods were provided: discussion groups, structured interviews, and a self-administered online questionnaire (Table 1).

**Table 1:** Participation methods and number of participants by method.

Participation method	Urban setting	Rural setting
A. Discussion groups	**91**	**53**
Intersectoral Participation Structure of XarxaSalut (professionals, local administration, and citizens)	13	11
Associations and Local Entities	10	13
Neighborhood Groups (Citizens and Local Administration)	17	–
Women in a vulnerable socioeconomic situation	7	7
People with Disabilities	10	3
Foreign Residents	8	–
Older adult/adults People	12	19
Young People	14	-
B. Structured interview	**83**	**46**
C. Online questionnaire	**68**	**15**
Total participants	**242**	**114**

Participant recruitment for any modality was carried out through social networks, social agents, and local community organizations, using convenience snowball and quota sampling. The sample size selected was based on representativeness and the saturation principle (65).

The discussion groups were designed based on the PROGRESS-Plus categories (66) (residential neighborhood, age, gender, employment status, educational level, nationality, and disability). These groups, facilitated by two experienced researchers, took place in various public administration spaces assigned for this purpose, and consisted of a total of 17 sessions lasting 120 min each. They were recorded with an audio recorder after obtaining informed consent of the participants, and corresponding field notes were created after their completion. The recordings were transcribed by the same team, maintaining verbatim transcription, and anonymizing their sources.

The structured interviews were conducted during community events with a large public turnout. A stand was set up in each municipality, introducing the project and offering the opportunity to participate on the spot. The online questionnaire was disseminated through a QR code included in dissemination materials and distributed through social networks, community networks, social agents, and community organizations.

All the participants were aged 18 and older and either lived or worked in the municipality being evaluated at the time of the study. They were all informed about the purpose of the study and provided explicit consent for their participation.

A detailed explanation of the sampling methodology and data collection process can be found elsewhere (57).

### 2.4 Variables, instruments, and tools

Sociodemographic data were collected from all study participants, regardless of the participation method, including gender, age, education level, employment status, degree of disability, nationality, neighborhood, and municipality of residence or work.

To comprehend the individual and collective perceptions of study participants regarding their place of residence or work, in terms of the physical, social, and economic factors of the environment, the Spanish adaptation of the Place Standard Tool (PST) called “Entornos de Vida” (EdV) (67) was utilized. This instrument, with a Cronbach’s Alpha reliability of 0.849 and an explained variance percentage of 0.842, facilitates the gathering of both quantitative and qualitative data describing the community’s perceptions and feelings about their place and how it can be improved to become a high-quality place.

The PST consists of 14 topics (Table 2), and through 14 main questions, it facilitates the assessment of a specified place. Each topic is rated on a scale from 1 to 10, with 1 being the worst possible score and 10 being the best. The evaluation of each municipality was conducted through discussion groups, structured individual interviews, and online self-administered methods. In the self-administered and individual interview modalities, participants rated each of the 14 tool topics from 1 to 10 and noted the rationale behind their scoring. In the discussion group format, each participant first individually rated each of the 14 EdV topics on a scale of 1 to 10 and briefly explained the reasoning for their rating. Subsequently, participants shared their scores and provided arguments for their assessments.

**Table 2:** Topics and main questions of the Place Standard Tool.

Topic		Main question
T1	Walking or cycling	How easy is it to move around and get to where I want to go?
T2	Public transport	What is public transport like in my place?
T3	Traffic and parking	How do traffic and parking affect how I move around my place?
T4	Streets and spaces	What are the buildings, streets and public spaces like in my place?
T5	Natural space	How easy is it for me to regularly enjoy natural space
T6	Play and recreation	How good are the spaces and opportunities for play and recreation in my place?
T7	Services	How well do services in my place meet my needs?
T8	Work and local economy	How active is the local economy in my place and are there good opportunities for work, volunteering, and training?
T9	Housing and community	How well do the homes in my place meet the needs of my community?
T10	Social interaction	How good is the range of opportunities which allow me to meet and spend time with other people?
T11	Identity and belonging	To what extent does my place have a positive identity that supports a strong sense of belonging?
T12	Feeling safe	How safe does my place make me feel?
T13	Care and maintenance	How well is my place looked after and cared for?
T14	Influence and sense of control	When things happen in my place how well am I listened to and included in decision-making?

Structured interviews and the self-administered online questionnaire facilitated the collection of both quantitative information (scores for the 14 topics) and qualitative information (rationales for the scores). In the discussion groups, both individual responses (scores and rationales) and comments generated by participants during the collective reflection on each of the 14 topics were also collected.

### 2.5 Data analysis

#### 2.5.1 Quantitative analysis

The Kolmogorov–Smirnov test was used to evaluate the normality of the sample distribution. Central tendency and dispersion measures were used to analyze quantitative variables. For qualitative variables, absolute and relative frequencies, expressed as percentages, were used. Non parametric tests (U de Mann–Whitney) were carried out to assess the relationship between rural and urban settings, and the 14 dimensions of EdV. Boxplots were used to describe settings based on the medians (M) and interquartile range (IQR) of each topic of the tool. The level of statistical significance was established at *p* < 0.05. The missing values for the dependent variables were left as missing values. All data were analyzed with Statistical Package for the Social Sciences (SPSS) version 25, Spanish.

#### 2.5.2 Qualitative analysis

The aim of the qualitative analysis was to understand residents’ perceptions of the physical and socio-economic aspects of their municipality based on the 14 areas evaluated in EdV. The qualitative analysis was conducted by adapting the thematic analysis model (68, 69) to the study’s objectives, using deductive categories centered on the 14 topics examined in the EdV tool. Data were coded to identify the themes that explained the central ideas regarding the perception of their municipality. Data saturation was reached by comparing patterns within discussion groups, comparatively with all groups and with interviews. Differences between resultant categories were resolved by a discussion and further clarification among the team members. We used NVIVO v1.7.1 software to manage the analytical process.

#### 2.5.3 Mixed method analysis

The two components of the study were analyzed concurrently, following the convergent-parallel approach (58). The quantitative analysis (QUAN) was conducted using the individual data collected through the place assessment instrument across all participation methods, while the qualitative analysis (QUAL) was carried out after conducting structured interviews and discussion groups, involving the coding of the participants’ observations. Subsequently, the results from the QUAL and QUAN components were integrated to gain a more comprehensive understanding of the communities’ perception of their municipality, compare them, and identify areas of convergence or divergence in the findings. No greater relevance was attributed to either aspect of the study; instead, the focus was on determining whether there was consistency between the qualitative and quantitative data. The analysis and interpretation are represented in a joint display (70).

### 2.6 Ethical aspects

This study was conducted in accordance with the Declaration of Helsinki and was approved by the ethics committee at the University of Valencia (reference no.1208176). Participants were fully informed and consented before participating, ensuring confidentiality and compliance with data protection regulations of both Spain and the European Union (Organic Law 3/2018 and General Data Protection Regulation (EU) 2016/679).

## 3 Results

### 3.1 Demographics

A total of 356 individuals participated in the study, with 242 from the urban area and 114 from the rural area. The proportion of women compared to men was higher (63.6% women) in all participation methods, except for the urban online questionnaire (50% men). In both municipalities, most participants came from discussion groups, followed by structured interviews and the online questionnaire (40.45, 36.24, and 23.31% respectively). The average age was 48.25 years in Denia (standard deviation (SD) = 17.13) and 52.57 in Yátova (standard deviation (SD) = 19.89), with a minimum age of 18 and a maximum age of 95. Regarding employment status, 2.5% were engaged in unpaid caregiving, 10.1% were unemployed, 10.7% were students, 26.1% were retired, and 49.7% were employed. In terms of education level, 12.6% had primary or lower education, 29.2% had completed secondary education, and 37.6% had higher education. Among the participants, 6.18% had a disability degree equal to or greater than 33%. Additionally, 12.69% of participants were foreigners (Table 3).

**Table 3:** Sociodemographic characteristics of the sample by participation method and setting (*n* = 356).

(*n*, %)	Urban setting (242, 67.98)			Rural setting (114, 32.02)		
	Discussion group	Structured interview	Online questionnaire	Discussion group	Structured interview	Online questionnaire
	91(37.60)	83 (34.30)	68 (28.10)	53 (46.54)	46 (40.35)	15 (13.16)
*Gender (n, %)*						
Female	61 (67.03)	58 (69.89)	33 (48.53)	38 (71.70)	25 (54.35)	10 (66.67)
Male	30 (32.97)	25 (30.12)	34 (50.00)	14 (26.41)	20 (43.48)	5 (33.33)
Other	–	–	1 (1.47)	–	–	–
Missing data	–	–	–	1 (1.89)	1 (2.17)	–
*Age (n, %)*						
18 to 35 years	27 (29.67)	14 (16.87)	26 (38.24)	7 (13.21)	18 (39.13)	5 (33.33)
36 to 50 years	20 (21.98)	18 (21.69)	23 (33.82)	7 (13.21)	6 (13.04)	7 (46.67)
51 to 65 years	25 (27.47)	32 (38.55)	14 (20.59)	14 (26.41)	18 (39.13)	2 (13.33)
66 or above	18 (19.78)	19 (22.89)	5 (7.35)	23 (43.40)	4 (8.70)	1 (6.67)
Missing data	1 (1.10)	–	–	2 (3.77)	–	–
*Education level (n, %)*						
Primary school or below	13 (14.29)	4 (4.81)	2 (2.94)	20 (37.74)	6 (13.04)	–
Secondary school uncomplete	15 (16.48)	18 (21.69)	3 (4.41)	11 (20.75)	17 (36.96)	8 (53.33)
Secondary school complete	39 (42.86)	26 (31.32)	21 (30.88)	9 (16.98)	9 (19.56)	–
Tertiary education or above	24 (26.37)	35 (42.17)	42 (61.76)	12 (22.64)	14 (30.43)	7 (46.67)
Missing data	–	–	–	1 (1.89)	–	–
*Employment status (n, %)*						
Student	18 (19.78)	2 (2.41)	10 (14.71)	2 (3.77)	3 (6.52)	3 (20)
Employed	31 (34.07)	45 (54.22)	43 (63.24)	17 (32.07)	31 (67.39)	11 (73.33)
Unemployed	12 (13.19)	5 (6.02)	8 (11.76)	7 (13.21)	4 (8.70)	–
Unpaid care	1 (1.10)	2 (2.41)	1 (1.47)	1 (1.89)	3 (6.52)	1 (6.67)
Retired	29 (31.87)	29 (34.94)	6 (8.82)	25 (47.17)	4 (8.70)	–
Missing data	–	–	–	1 (1.89)	1 (2.17)	–
*Degree of Disability (n, %)*						
33% or below	81 (89.01)	78 (93.98)	67 (98.53)	47 (88.68)	46 (100)	14 (93.33)
33% or above	10 (10.99)	5 (6.02)	1 (1.47)	5 (9.43)	–	1 (6.67)
Missing data	–	–	–	1 (1.89)	–	–
*Nationality (n, %)*						
Spanish	68 (74.72)	68 (81.93)	43 (63.24)	52 (98.11)	44 (95.65)	14 (93.33)
European Union	7 (7.69)	11 (13.25)	2 (2.94)	1 (1.89)	-	1 (6.67)
Not European Union	14 (15.39)	4 (4.82)	–	–	2 (4.35)	–
Missing data	2 (2.20)	–	23 (33.82)	–	–	–

### 3.2 Quantitative results

The results of the quantitative part of the study showed that the worst-rated themes in both contexts were *Public Transport* as well as *Work and Local Economy*. In the urban area, there were also negative ratings for *Traffic and Parking, Housing and Community*, and *Care and Maintenance*. The areas that received the highest overall scores were *Identity and Belonging, Natural Spaces, Streets and Spaces, Social Interaction*, and *Services*. In the urban context, the perception of safety*, Feeling Safe*, was also highly rated. In the rural area, *Walking or Cycling, Traffic and Parking, Housing and Community*, and *Influence and Sense of Control* were also positively evaluated (Table 4).

**Table 4:** Medians corresponding to the quantitative evaluation of settings based on sociodemographic characteristics of participants and rated EdV topic.

Sociodemographic characteristics								Topic EdV								
		T1	T2	T3	T4	T5	T6	T7	T8	T8	T9	T10	T11	T12	T13	T14
Rural setting		Median														
Sex	Female	6.00	4.00	4.00	7.00	7.00	6.00	7.00	4.00	5.00	7.00	8.00	7.00	6.00		6.00
	Male	6.50	4.00	5.00	7.00	7.00	6.00	7.00	5.00	5.00	7.00	8.00	8.00	5.00		6.00
Age group	18–35	7.00	4.00	4.00	7.00	7.00	6.00	7.00	5.00	5.00	6.00	8.00	7.00	6.00		6.00
	36–50	5.00	3.00	3.00	6.00	7.00	6.00	7.00	4.00	5.00	6.00	8.00	8.00	5.00		6.00
	51–65	7.00	4.00	5.00	7.00	7.00	6.00	7.00	4.50	5.00	7.00	8.00	7.00	5.00		5.00
	>65	7.00	5.00	5.00	7.00	6.00	6.00	8.00	4.00	6.00	8.00	8.00	7.50	6.00		7.00
Educational level	Primary	6.50	5.00	5.00	7.50	8.00	7.00	8.00	4.00	4.50	8.00	9.00	8.00	7.00		8.00
	Second Incomplete	6.00	3.00	3.00	7.00	6.00	6.00	7.00	5.00	4.00	7.00	8.00	7.00	5.00		5.00
	Secondary	6.00	4.00	4.00	7.00	7.00	5.00	7.00	4.00	5.00	6.00	8.00	7.00	6.00		6.00
	Tertiary	6.00	3.00	5.00	7.00	7.00	6.00	7.00	5.00	6.00	7.00	7.00	8.00	6.00		6.00
Employment situation	Student	6.00	4.50	4.00	7.00	7.00	6.00	8.00	5.00	5.00	5.50	8.00	8.00	6.00		5.00
	Employed	6.00	3.00	4.00	7.00	7.00	6.00	7.00	5.00	5.00	7.00	8.00	7.00	5.00		6.00
	Unemployed	6.00	3.50	4.00	7.00	7.00	5.00	7.00	3.00	3.00	5.00	8.00	6.00	5.00		5.00
	Unpaid care	5.00	5.00	2.00	5.00	8.00	6.00	7.00	3.50	5.00	5.00	7.00	7.50	5.00		7.00
	Retired	7.00	5.00	5.00	7.00	8.00	6.00	8.00	4.00	5.00	8.00	8.00	8.00	6.00		6.00
Disability	≥33%	7.00	4.00	4.00	8.00	8.00	8.00	8.00	5.00	4.50	8.00	9.00	8.00	5.00		7.00
	<33%	6.00	4.00	4.00	7.00	7.00	6.00	7.00	5.00	5.00	7.00	8.00	7.00	5.50		6.00
Nationality	Spanish	6.00	4.00	4.00	7.00	7.00	6.00	7.00	5.00	5.00	7.00	8.00	7.00	5.00		6.00
	European	7.00	4.00	5.00	8.00	7.50	7.00	8.00	4.00	5.50	8.00	9.00	7.50	5.00		6.00
	Non-EU	7.00	4.00	4.00	8.00	8.00	6.00	8.50	4.00	3.50	6.00	9.00	9.00	7.00		8.00
Rural setting		Median														
Sex	Female	7.00	5.00	7.00	8.00	8.00	5.00	7.00	5.00	7.00	7.00	9.00	6.75	6.00		7.00
	Male	7.00	5.00	7.00	7.00	8.00	6.00	7.00	4.00	6.00	7.00	9.00	7.00	6.00		6.00
Age group	18–35	7.00	6.00	8.00	8.00	8.00	6.00	7.00	5.00	8.00	7.00	9.00	6.50	6.00		5.00
	36–50	8.00	4.50	8.00	8.00	9.00	6.50	7.50	4.50	6.00	7.00	9.00	7.00	7.00		7.00
	51–65	7.00	5.00	8.00	8.00	8.00	6.00	6.00	4.50	7.00	8.00	9.00	6.50	6.00		8.00
	>65	5.00	4.00	4.75	7.00	6.50	4.00	5.00	3.00	5.00	5.00	8.00	6.00	5.00		7.00
Educational level	Primary	5.00	5.00	5.00	7.00	7.00	4.50	5.00	3.00	6.00	6.00	7.00	5.50	6.00		6.50
	Second incomplete	7.00	5.00	7.00	8.00	9.00	6.00	7.00	4.00	7.00	8.00	10.00	6.75	5.50		6.50
	Secondary	7.00	5.00	8.00	8.00	9.00	5.00	7.00	5.00	7.00	6.00	9.00	7.00	6.00		8.00
	Tertiary	8.00	4.00	8.00	8.00	8.00	6.00	7.00	5.00	6.00	7.00	9.00	7.00	6.00		7.00
Employment situation	Student	7.00	7.00	8.00	8.00	8.00	6.50	6.50	5.50	7.00	8.00	9.50	7.00	7.00		8.00
	Employed	8.00	5.00	8.00	8.00	8.00	6.00	7.00	4.00	7.00	7.00	9.00	7.00	6.00		7.00
	Unemployed	7.00	5.00	8.00	8.00	9.00	6.00	7.00	5.00	8.00	9.00	9.00	6.00	4.00		5.00
	Unpaid care	9.00	5.00	8.00	10.00	10.00	7.00	6.00	6.00	7.00	8.00	10.00	5.00	6.00		2.00
	Retired	5.00	5.00	5.00	7.00	7.00	5.00	5.00	4.00	5.00	6.00	8.00	6.50	5.00		8.00
Disability	≥33%	5.50	4.00	8.50	7.00	7.50	5.50	5.50	3.50	8.00	7.00	8.50	5.50	6.50		7.50
	<33%	7.00	5.00	7.00	8.00	8.00	6.00	7.00	4.00	6.00	7.00	9.00	7.00	6.00		7.00
Nationality	Spanish	7.00	5.00	7.00	7.50	8.00	6.00	7.00	4.00	7.00	7.00	9.00	7.00	6.00		7.00
	European	8.50	5.00	5.50	6.50	10.00	6.00	6.00	3.50	7.50	10.00	5.50	6.50	4.00		2.00
	Non-EU	8.50	10.00	7.50	9.00	9.00	6.50	10.00	4.50	5.50	10.00	10.00	10.00	9.00		6.50

Medians are ordered by color from red to green, red being the lowest score and green corresponding to the highest score.

Regarding scores by population groups, in the urban context, people who were unemployed and those engaged in unpaid caregiving rated more negatively, while those with primary education and disabilities gave more positive evaluations. The rural context received less favorable ratings from individuals aged over 65, retirees, and those with primary education, while it was more positively evaluated by foreign individuals from outside the European Union.

When conducting a comparative analysis between the urban and rural contexts (Table 5), it was evident that the perceptions in the rural setting showed a more favorable assessment in most topics, except for *Work and Local Economy*, which received a higher rating in the urban context. However, in *Play and Recreation*, *Services*, *Social Interaction*, and *Feeling Safe*, similar scores were obtained in both places. It is worth noting that five themes showed highly significant differences (with values of *p* < 0.01) between the two settings.

**Table 5:** Comparison of medians by setting and EdV topic.

Topic	Setting (*n*)	Median (IQR)	Min-Max	*p*
Walking or cycling	Urban (237)	6.00 (4)	1–10	0.018*
	Rural (113)	7.00 (3)		
Public transport	Urban (236)	4.00 (4)	1–10	0.002*
	Rural (110)	5.00 (3)		
Traffic and parking	Urban (237)	4.00 (3)	1–10	0.000*
	Rural (111)	7.00 (4)		
Streets and spaces	Urban (237)	7.00 (3)	1–10	0.000*
	Rural (112)	8.00 (2)		
Natural space	Urban (237)	7.00 (3)	1–10	0.000*
	Rural (111)	8.00 (3)		
Play and recreation	Urban (240)	6.00 (3)	1–10	0.827
	Rural (109)	6.00 (3)		
Services	Urban (239)	7.00 (2)	1–10	0.596
	Rural (111)	7.00 (4)		
Work and local economy	Urban (238)	5.00 (2)	1–10	0.581
	Rural (109)	4.00 (3)		
Housing and community	Urban (240)	5.00 (3)	1–10	0.000*
	Rural (110)	7.00 (3)		
Social interaction	Urban (239)	7.00 (3)	1–10	0.024*
	Rural (110)	7.00 (3)		
Identity and belonging	Urban (238)	8.00 (3)	1–10	0.000*
	Rural (111)	9.00 (3)		
Feeling safe	Urban (239)	7.00 (4)	1–10	0.056
	Rural (109)	7.00 (3)		
Care and maintenance	Urban (238)	5.00 (3)	1–10	0.280
	Rural (111)	6.00 (3)		
Influence and sense of control	Urban (236)	6.00 (4)	1–10	0.147
	Rural (108)	7.00 (4)		

^*^p < 0.05.

### 3.3 Qualitative results

In the qualitative analysis, key ideas explaining each of the 14 EdV themes were identified. In the urban context, 48 categories and two new emerging themes, Tourism and Accessibility, were established. In the rural setting, they were grouped into 39 categories. Table 6 includes the categories and descriptive labels for each EdV topic in both contexts.

**Table 6:** Themes and descriptive labels for each assessed topic of EdV in rural and urban settings.

Topic	Categories and description	
	*Urban setting*	*Rural setting*
T1	**Barriers to Walking** Condition of sidewalks and urban infrastructure that hinders pedestrian movement.	**Facilitating and hindering elements for walking** street and sidewalk features, infrastructure, and architectural barriers.
	**Road Safety** Lack of safety for pedestrians and cyclists.	**Road Safety** Lack of safety for cyclists.
	**Cycling** Characteristics of the cycling network and cycling culture.	**Cycling** Characteristics of the cycling network and cycling culture.
T2	**Connections** Inefficient connections between essential facilities, neighborhoods, and transportation modes.	**Connections** Lack of connection to essential facilities, other municipalities, and transportation modes.
	**Prices** Transportation costs and discount systems.	**Accessibility** Barriers to accessing transportation services.
	**Information and Service** Access to transportation information, such as schedules, and quality of transportation services.	**Transportation Service** Limited transportation availability and frequency.
	**Where It Does not Take You** Places that cannot be reached by public transportation.	
T3	**Parking** Conditions of public and private parking.	**Parking** Ease of parking.
	**Pedestrianization of Streets** Experiences related to pedestrian zones.	
	**Traffic** Negative perception of the impact of traffic on well-being.	**Traffic** Low traffic and impact of car overuse.
T4	**Shade** Shaded areas as a requirement on streets and public spaces.	**Lack of Facilities** Mobility aids, lighting, and shade.
	**Pedestrian Experience** Walking experience on city streets and in public spaces.	**Aesthetics** Aesthetic perception of public places.
T5	**Natural Areas in the Vicinity of the City** Natural spaces, facilities, and highlighted features.	**Natural Areas Outside the Town** Natural zones and the Mijares mountain range.
	**Urban Parks** Characteristics of parks within the urban area.	**Natural Areas Inside the Town** Characteristics of municipal parks.
	**Beaches** Characteristics of the municipality’s beaches, and issues with algae.	
	**Demands** Shortages in spaces, facilities, and maintenance of natural areas.	
T6	**Positively Valued Activities** Characteristics of leisure activities and target population.	**Spaces and Activities** Characteristics of the leisure space and activity offerings.
	**Demand for Activities** Demand for specific activities.	**Demands for Play and Leisure** Requests for spaces and activities by population groups.
	**Cinema** Access difficulties to movie theaters.	
	**Sports** Characteristics of the municipality’s sports offerings.	
	**Young People** Lack of leisure offerings for young people.	
T7	**Public Services** Accessibility to administrative procedures and lack of public services.	**Public Services** Characteristics of the public service offerings.
	**Healthcare** Demands for improvement in the quality of public healthcare services.	**Demand** Demand for new services and improvement of existing ones.
	**Local Commerce** Characteristics of the local commercial offerings.	**Local Commerce** Characteristics of the local commercial offerings.
T8	**Employment Opportunities** Unemployment, job difficulties, and job insecurity.	**Employment Opportunities** Possibility of employment or creation of new jobs.
	**Local Economy** Characteristics of local businesses and types of industries.	**Local Economy** Characteristics of the local economic activity.
	**Education and Training** Supply and demand for training in the local economic sectors.	**Impact of Local Unemployment** Opportunities for employment outside the municipality.
T9	**Housing Rental** Characteristics of the rental housing market.	**Housing Supply** Characteristics of available rental or purchase housing.
	**Housing Access** Barriers preventing access to housing.	**Owner-Occupied Housing** Types of local owner-occupied housing.
		**Vacant Housing** Characteristics and causes of vacant housing.
T10	**Social Spaces** Places that facilitate gatherings and demands for new spaces.	**Social Spaces** Demand for spaces for social interaction.
	**Promotion of Social Contact** Groups and organizations that promote social contact.	**Promotion of Social Contact** Local associations.
	**Relationship Challenges** Lack of meeting places, language barriers, consumption-related leisure.	**Activities** Activities that promote social contact.
	**Loneliness** Groups and individuals with a perception of loneliness.	**Childhood and Adolescence** Demand for social interaction spaces and activities during winter and weekends.
T11	**Integration Challenges** Barriers that hinder a sense of identity and belonging to the municipality.	**Integration Challenges** Negative aspects related to the sense of belonging and integration.
	**Identity Factors** Aspects that contribute to a sense of belonging to the municipality.	**Identity Elements** Factors that facilitate the feeling of belonging to the municipality.
T12	**Threats to Personal Safety** Experiences of fear and danger perception.	**Disruptions** Events that threaten security.
	**Police** Presence and perception of police activity.	**Police** Perception of police absence.
	**Perception of Safety** Factors that promote the perception of personal safety.	**Perception of Safety** Factors related to personal safety.
T13	**Municipal Actions** Activities perceived as the responsibility of municipal services that are not being carried out or need improvement.	**Municipal Actions** Activities perceived as the responsibility of municipal services that are not being carried out or need improvement.
	**Garbage and Recycling** Municipal and community compliance with garbage collection and recycling.	**Garbage and Recycling** Garbage and recycling collection system and perception of municipal and community compliance.
	**Natural Spaces** Perception of the care and maintenance of both urban and out-of-town natural areas.	**Cleanliness** Perception of the cleanliness level of public space.
	**Activities Generating Litter** Public drinking and street markets.	
	**Lack of Civic Responsibility** Neighbor behavior related to a lack of care and maintenance.	**Lack of Civic Responsibility** Neighbor behavior related to a lack of care and maintenance.
T14	**Participation Channels** Characteristics of the known local participation channels.	**Participation Channels** Characteristics of the known local participation channels.
	**Challenges in Participating** Cultivation of a participation culture and efforts to encourage involvement.	**Challenges in Participating** Barriers to engagement.
	**Influence and Participation in Decision-Making** Perceived capacity in local decision-making.	**Influence and Participation in Decision-Making** Perceived capacity in local decision-making.
E**	**Tourism** Impact on employment, housing, and living conditions during the summer.	
	**Accessibility** Economic, informational, architectural, and digital difficulties and barriers.	

^*^Themes evidenced in discussion groups and structured interviews.

^**^Emerging Themes.

Below, the arguments that make up the perceptions of the participants regarding each of the 14 EdV themes, both in the rural and urban settings, are summarized. The Supplementary material (Supplementary Tables S2,S3) contains verbatim quotes that illustrate the results from discussion groups and structured interviews.

#### 3.3.1 Walking or cycling

The positive perception of walkability in the city was described as influenced by sidewalk characteristics, the lack of shade, and the absence of amenities, such as benches and public restrooms. Mobility barriers were associated with disabilities and caregiving responsibilities. In the rural setting, the ability to walk or cycle everywhere was positively valued. Negative aspects were linked to narrow sidewalks, lack of amenities, and architectural barriers.

Being able to cycle was positively rated due to the climate and the city’s characteristics, its size, and the absence of steep slopes. However, barriers were identified, such as a lack of bike lanes, poorly maintained or non-existent bike lanes, and a limited “bike culture.” In both settings, there was a perceived insecurity when walking and cycling that related to the lack of compliance with the traffic regulations and to the hegemony of motor vehicles over pedestrians and cyclists.

#### 3.3.2 Public transport

This topic was rated very negatively in both settings by all social groups. In the city, it was associated with a lack of connectivity to other municipalities, such as the provincial capital or nearby towns, basic facilities like hospitals, shopping centers, schools, and natural spaces, as well as between different neighborhoods within the city. Additionally, there were calls for price discounts for specific groups to lower the cost, which was considered expensive for the service provided. Lack of information about bus frequencies, stops, routes, and schedules was also reported.

The residents of the rural setting indicated that public transport was virtually nonexistent. Although there is a local bus and a regional train station in a nearby municipality, issues related to accessibility, mobility, ticket cost, and the lack of connectivity between modes of transportation, basic amenities, and the upper part of the village were described.

#### 3.3.3 Traffic and parking

In the urban context, this theme, that boasts a dual composition, was characterized by a general lack of free and public parking, and the highly negative impact of traffic due to congestion, noise pollution, and pollution, primarily occurring in the summer. It was emphasized that, although the city’s layout makes it easy to walk everywhere, there is a prevailing car culture. Street pedestrianization was considered effective in improving the city’s quality of life but faced opposition from a segment of the population. In the rural setting, there was a positive appreciation for being able to park anywhere in the village and the low traffic levels. Negative perceptions were based on excessive car usage for short trips.

#### 3.3.4 Streets and spaces

The city was described as a pleasant and aesthetically pleasing environment, despite the disparity in architectural styles. Iconic places like the castle, the Les Rotes promenade, and the city center were positively highlighted. It was considered essential for streets and public spaces to have benches and shade during the summer. The rural municipality positively evaluated its streets and called for better lighting, mobility facilitators, trees, and improved maintenance.

#### 3.3.5 Natural spaces

The Montgó Massif Natural Park and the beaches of Denia were identified as very close and accessible natural spaces whose care was improved in recent years. Beach cleanliness generated controversy due to the presence of posidonians aquatic plant, which accumulates on the beaches during the winter, preventing their degradation but also limiting enjoyment beyond summertime and incurring high cleaning costs. Despite the favorable evaluation of urban parks, there is a need to improve cleaning efforts. The need for more natural spaces, trees, and cool, shaded areas was also emphasized. On the other hand, Yátova is surrounded by highly valuable natural spaces, such as the Martés mountains and the Mijares, Magro, and Juanes rivers, and it has several parks within the inner city. The local government’s efforts in accessibility and conservation of these spaces were highlighted.

#### 3.3.6 Play and recreation

The city has numerous community resources, including associations, a senior center with a varied range of activities, a cultural center with an extensive program, and a highly rated youth center. However, there was a perceived need for more cultural activities, healthy and free leisure alternatives for young people, additional public facilities, and a program for free access to sports. In the rural area, there are sports facilities and activities, a senior center, and it is positively valued that children continue to play outdoors. However, there is a need to offer leisure alternatives for both young and older residents, promote cultural activities, and establish programs during the winter months and on weekends.

#### 3.3.7 Services

In the city, it is considered that there is a wide range of services, and there is a positive appreciation for having a sufficient variety of shops and offices of all government administrations. However, there is a demand for more public services such as universities, daycare centers, and improvements in healthcare services. In the rural context, it is considered that there is sufficient commerce to acquire basic food products, albeit at higher prices, and the senior day center is highly valued. However, there is a demand for improvements in healthcare services and quality restaurants.

#### 3.3.8 Work and local economy

In the urban setting, this theme is negatively evaluated due to precarious and seasonal employment, mostly linked to the tourism sector. Additionally, there are mentions about the difficulty in accessing quality jobs for people aged over 45 and young individuals, as well as challenges in creating new local businesses. There are identified latent opportunities in various sectors, such as the nautical and the environment industries, with a demand for specialized training related to these fields. In the rural context, this topic is negatively evaluated due to the lack of local businesses and employment opportunities, which lead some people to migrate to other municipalities. However, potential business areas, like restaurants, tourism and the environment, are positively identified.

#### 3.3.9 Housing and community

Housing is identified as one of the major problems in the city. Tourist rentals drive up housing costs and make long-term renting difficult. Young people and individuals with fewer resources struggle to access housing in good conditions. On the other hand, in the village, the majority of the population owns their well-maintained homes. However, there is a perceived shortage of housing in good conditions, both for rent and purchase, despite having a significant stock of vacant housing.

#### 3.3.10 Social interaction

In the city, social interaction is facilitated by the network of associations and by the senior center and youth center. However, barriers to interaction are described, such as a lack of free activities, spaces for adults, and the Valencian language. Loneliness is perceived as an issue among older people and foreign residents. In the rural setting, social interaction is primarily facilitated by the existence of multiple associations, and there is a demand for spaces and activities that promote relationships for children and teenagers.

#### 3.3.11 Identity and belonging

In the urban setting, the construction of a positive identity was linked to the privileged characteristics of the natural environment and the openness of its people, with a greater sense of belonging among those born in Spain. A weaker connection and sense of identity were associated with belonging to a foreign community and the challenges of integration. In the rural area, a stronger sense of identity was linked to the existence of community organizations, as well as a positive perception of belonging to Yátova and ease of integration. However, an opposing perception was also noted, where integration is not seen as a straightforward process.

#### 3.3.12 Feeling safe

Safety is addressed in two more areas (Walking or Cycling and Streets and Spaces) and related to road safety and street lighting. Most participants described the city as safe, although concerns were raised about the lack of police presence in summertime and incidents of theft and vandalism. Women expressed feeling unsafe when walking alone at night. In contrast, the rural setting is perceived as very peaceful and safe, with few incidents. The widespread criticism focuses on young people, who are blamed for vandalism, and the ambivalent perception of the absence of local police.

#### 3.3.13 Care and maintenance

This theme is related to the topics Natural Spaces and Streets and Spaces. In the city, the municipality’s efforts in maintaining natural spaces are negatively evaluated, while the cleaning and maintenance of urban spaces are viewed favorably. It is suggested that the lack of civility is the source of problems in this field. Additionally, inadequate use of trash and recycling containers, numerous dog feces in the streets, and specific areas being particularly dirty due to weekend binge drinking and the municipal market, are reported. In the village, the cleaning efforts of municipal services are acknowledged, and the responsibility for the problems in this regard is attributed to the citizens.

#### 3.3.14 Influence and sense of control

This topic was argued along three lines: the existing channels of participation, the difficulties in participating, and the perception of the capacity to influence municipal decision-making. In both contexts, some confusion was observed between participation and purely informational channels or consultative processes. Associationism and social networks were identified as channels of participation. Regarding difficulties, the “lack of a culture of participation” was mentioned, and in terms of the perception of influence capacity, there were reports of a lack of listening and information from the local government, as well as a low capacity to influence local policies. The participation channels described in the urban context were social networks, the municipal app, and neighborhood councilors, while in the rural context, channels were more related to the accessibility of the local government, such as participating in municipal meetings or having direct access to the mayor.

#### 3.3.15 Emerging themes in the urban context

Throughout the study, two themes were identified that cut across all topics and population groups: tourism and accessibility. Tourism had a significant and negative impact on housing, employment, and living conditions during the summer. Issues related to access to information, resources, activities, and public and private services were linked to the digital divide, lack of economic resources, information channels, and architectural barriers.

#### 3.4 Integration of qualitative and quantitative findings

The joint display developed for this mixed methods research is a combination of a side-by-side display and a comparing-result display (70), as it presents a visual representation of the results that combines both quantitative and qualitative data (Table 7). Congruencies, discrepancies between municipalities, and interpretation of the results are also included. In the left column, there is a graph showing the medians and interquartile ranges (IQR) of the scores obtained for each EdV theme, with blue representing the urban setting and orange representing the rural area. In the right column, the qualitative results and interpretation of the meaning of the results are presented. Key points shared by both contexts are highlighted in green, those exclusive to the urban setting are indicated in blue, and those exclusive to the rural context are highlighted in orange. The interpretation of the main results is presented in black.

**Table 7:** Joint display of quantitative and qualitative results.

Quantitative scores by EdV theme and setting (Medians, IQR, and *p*)	Key qualitative results shared, by context and interpretation
*p-value < 0.05* 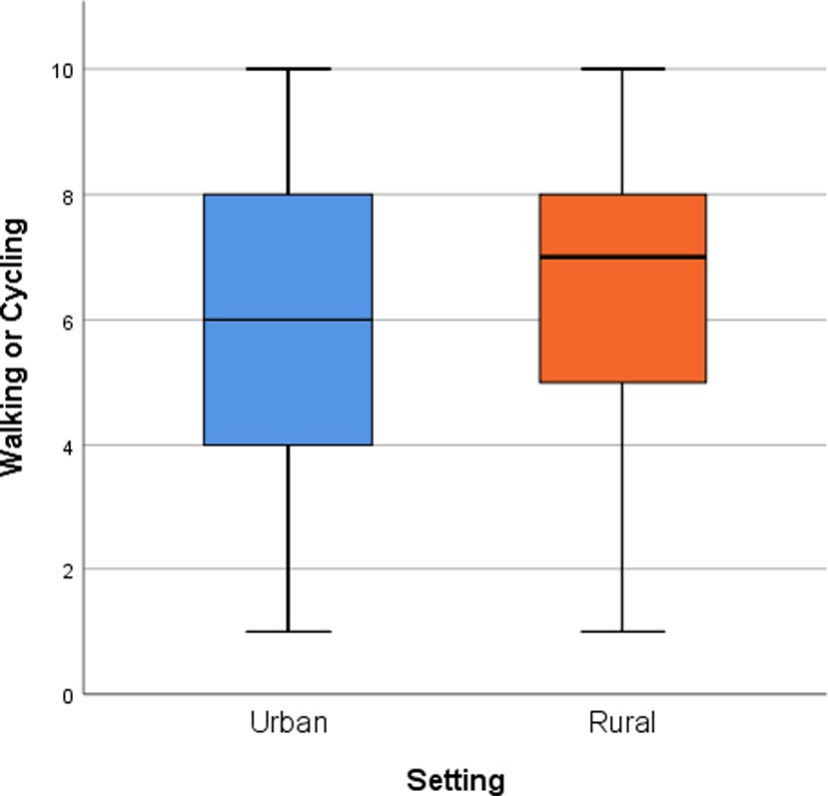	**Common results:** Walkable municipalities everywhere. Lack of bike lanes, road safety needs improvement, and bike culture needs to be promoted. Mobility barriers. Lack of amenities: shade and benches.
	**Urban context:** Lack of amenities, such as public toilets, benches, shaded areas. Road insecurity. Car hegemony.
	**Rural context:** Mobility and caregiving challenges: narrow sidewalks, need to walk on the road, architectural barriers.
	**Interpretation**: The more negative assessment in the urban context is influenced by not having a developed cycling network and a culture similar to other cities in the province. Also, traffic is perceived as a source of insecurity for walking or cycling. Architectural barriers that hinder mobility and caregiving-related activities prevail in the rural context, with a higher perception of walkability, even on the road, influenced by low road traffic.
*p-value < 0.05* 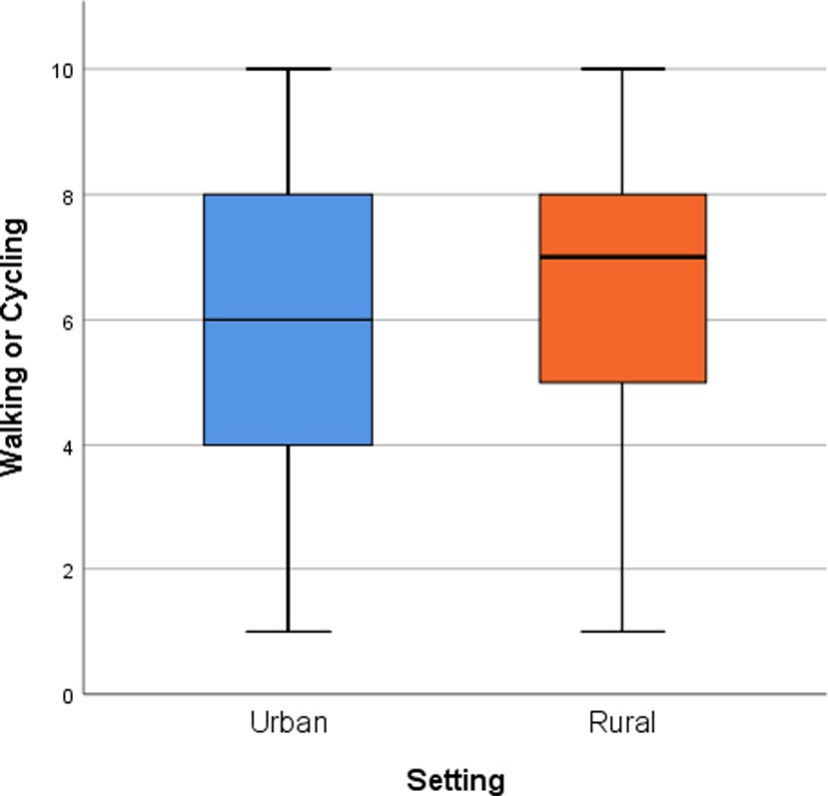	**Common results:** Poor connectivity to basic facilities (hospital, schools) and other municipalities. No price discounts for specific groups.
	**Urban context:** Lack of connectivity to other municipalities, between neighborhoods, with the shopping center, and natural areas. Scarce and inaccessible information about the service: frequencies, stops, routes. High prices.
	**Rural context:** Train station connected by bus. Lack of coordination between transportation modes (train-bus). Transportation not accessible to people with reduced mobility.
	**Interpretation**: In the rural context, the lack of access to better public transportation is often considered normal, leading to a greater reliance on private vehicles. In contrast, in the urban setting, transportation is more often a public service intended to meet the mobility needs of the population. As a result, the quality of this service is evaluated more critically, highlighting aspects such as the disconnection of basic infrastructure and a greater impact on various population groups, such as migrants, older adults, and women.
*p-value < 0.05* 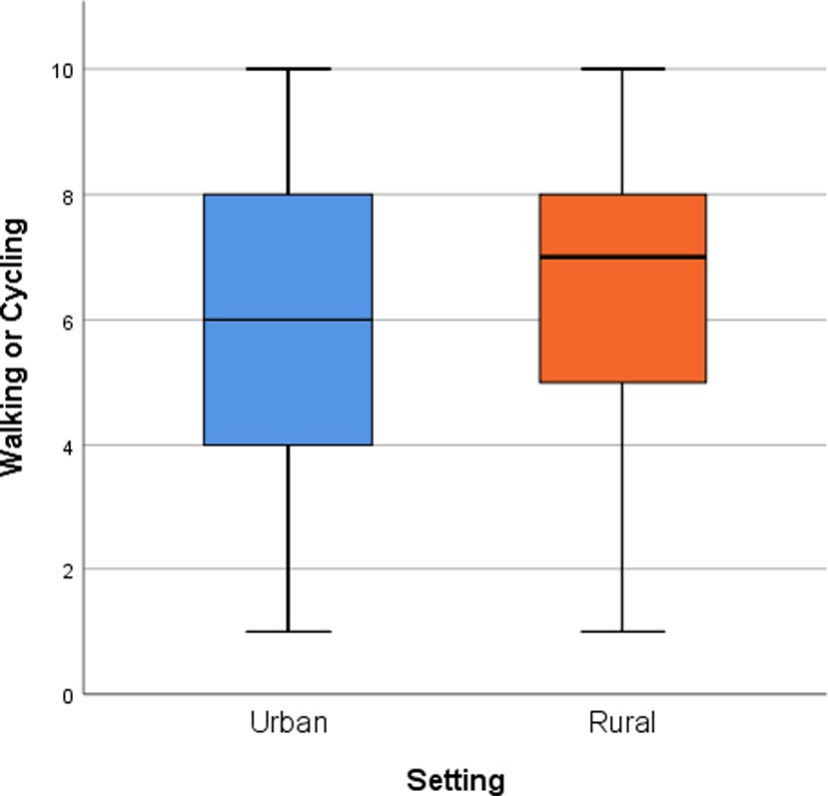	**Common results**: Car culture prevail over walking for commuting. Perception of lack of road safety in shared areas with vehicles.
	**Urban context**: Lack of free public parking. Deficiency in discouraging parking facilities that promote walking. Lack of public transport. Traffic jams, pollution, and noise. Effective and contentious street pedestrianization experiences.
	**Rural context:** Low traffic. Public parking without difficulty. Overuse of cars for short trips.
	**Interpretation**: The significant disparity between both contexts lies in the noticeable influence of heavy traffic and insufficient parking in the urban context, in contrast to the availability of space and limited presence of vehicles in the rural setting. This theme also influences the assessment of public transport, as its inadequacy is identified as a factor that encourages excessive use of private cars. Additionally, in walking or cycling, it is related to the perceived road insecurity in both settings. The dispersion of rural scores is linked to the favorable perception of those who can park close to their homes and the negative opinion that in the past, there were fewer vehicles on the streets, and they were safer.
*p-value < 0.05* 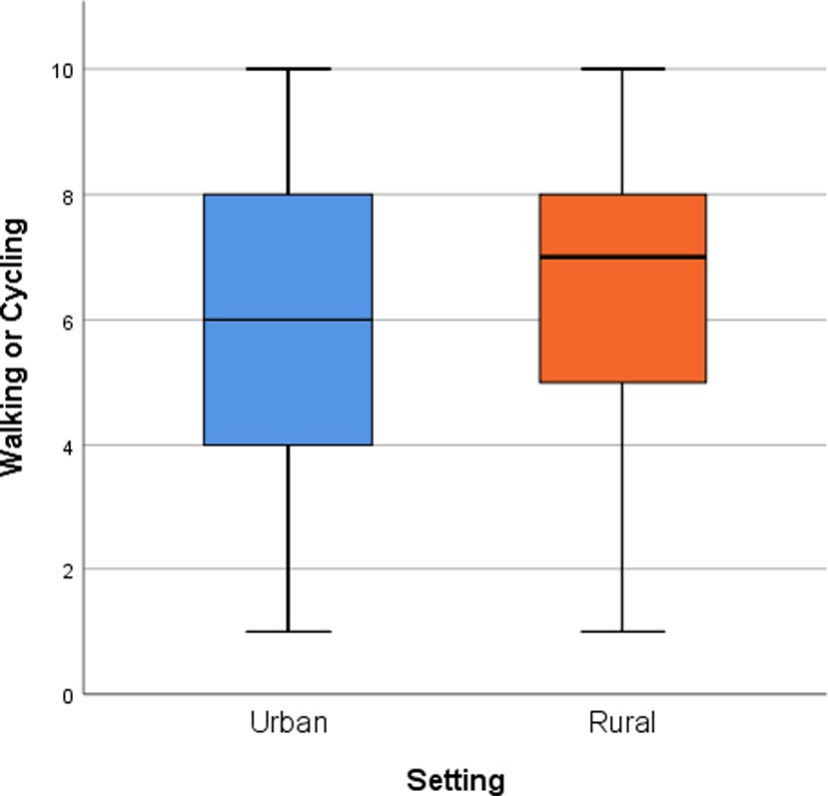	**Common results:** Streets and public spaces are considered pleasant places to walk through. There is a demand for shaded areas and benches for sitting during summertime. Street cleanliness needs improvement. There are architectural barriers that hinder transit.
	**Urban context:** Large differences between areas related to the heterogeneous urban and architectural model contribute to the creation of aesthetically unattractive areas. Heat and availability of shade, along with traffic density, determine the assessment of street transit. There is a noticeable demand for improving lighting in various areas.
	**Rural context**: Pleasant and beautiful village. Demand for trees on the streets and improvement in cleanliness. The difference in elevation between one part and another of the village hinders transit.
	**Interpretation**: The planning and architectural heterogeneity with differences between neighborhoods contributes to greater variability in scores in the urban setting, whereas there is a homogeneous model in the rural context. In the urban context, street cleanliness, lack of shade, and road traffic have a more negative impact. The perception of this dimension is strongly influenced by the care and maintenance aspect, where a lack of cleanliness is the main reason for lower scores.
*p-value < 0.05* 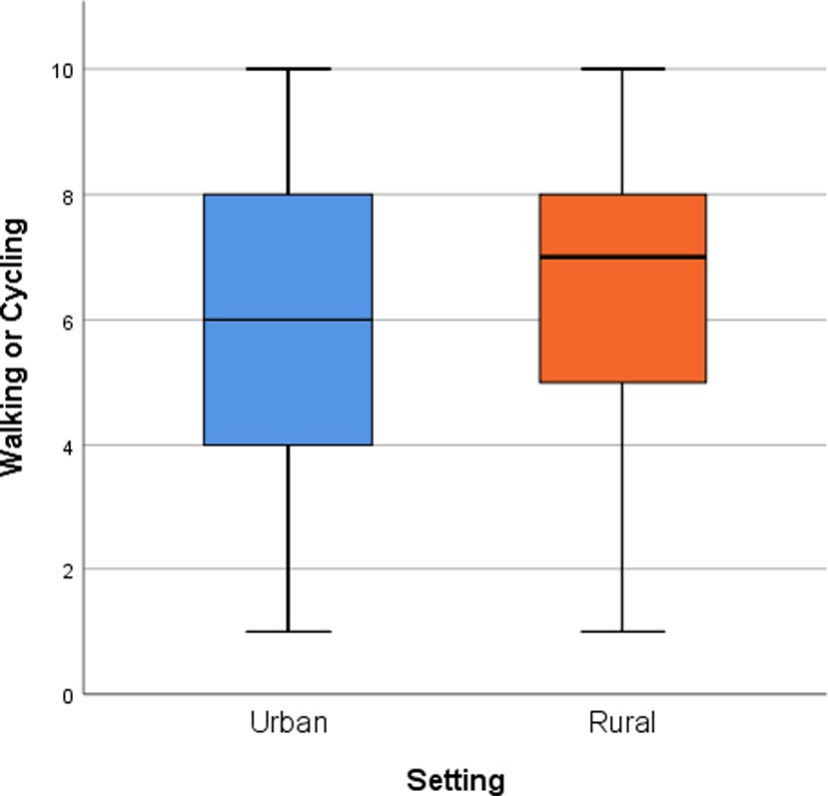	**Common results:** Nearby natural spaces are an important asset in both contexts. The involvement of local governments in their care and maintenance is emphasized. There is a demand for more wooded areas and cool, shaded spaces.
	**Urban context:** Beaches generate controversy due to the marked difference in their quality during the summer when they are highly appreciated and in winter due to the presence of seagrass. Accessible and adapted beaches are highly valued. There is a demand for more urban parks that are better cared for and maintained.
	**Rural context**: Natural spaces in the municipality’s surroundings are accessible and adapted and are highly valued. The municipality has few parks, but they are well appreciated. There are public projects for environmental conservation and enhancement of specific natural environments.
	**Interpretation**: Positive appreciation is related in the urban context to the quantity and quality of urban parks, while in the rural context, it is associated with the natural spaces surrounding the municipality. The lack of care and maintenance of parks and beaches contributes to the negative assessment of the city. The dispersion toward higher scores in the rural context is linked to recent local investment projects in the conservation and enhancement of the natural environment.
*p-value > 0.05* 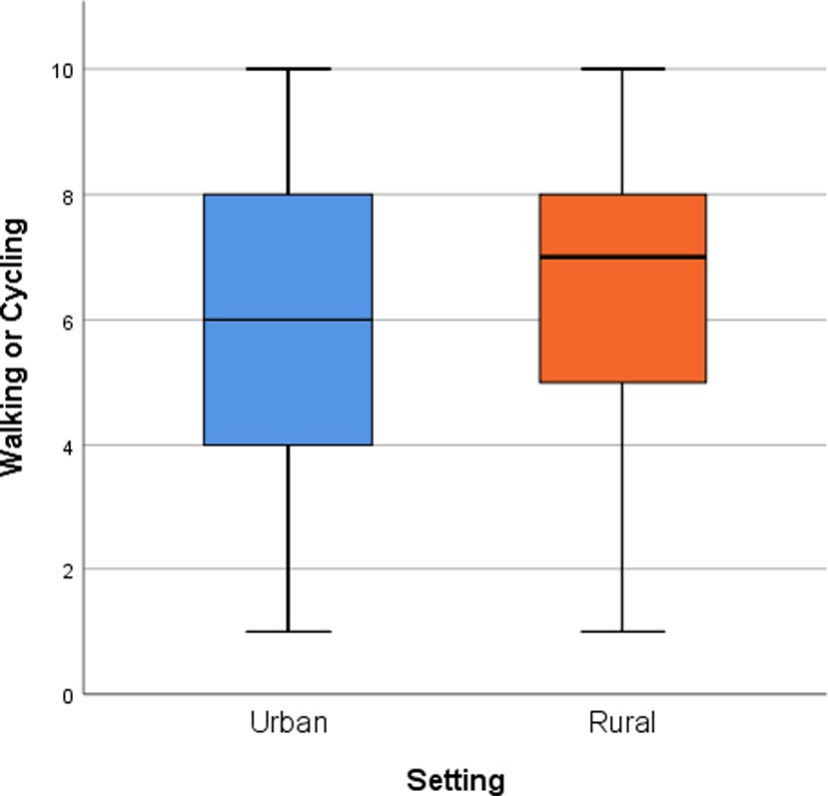	**Common results**: There are associations and centers for seniors and young people with scheduled activities. There is a demand for more cultural and healthy leisure activities for young people, as well as free options. There is also a call for more play areas for children and improved access to sports. The lack of activities during the winter and on weekends is noted.
	**Urban context:** There is a demand for spaces such as theaters, cinemas, and access to the shopping center. Free leisure activities are requested. A significant portion of young people’s leisure activities are related to bars, pubs, and nightclubs.
	**Rural context:** Activities related to natural spaces. Leisure alternatives in nearby towns. Children continue to play in the street.
	**Interpretation**: Play and recreation are related to cultural, sports, and social interaction-promoting activities. In both contexts, it is recognized that children and adolescents are particularly vulnerable groups due to the lack of spaces and activities. There is a perceived lower availability of activities in winter and on weekends, attributed to the tourist-focused leisure approach in the city and the low population density and limited recreational offerings in the rural municipality. In the city, the absence of free activities for adults not linked to consumption stands out, while in the rural municipality, children are encouraged to play in the street.
*p-value > 0.05* 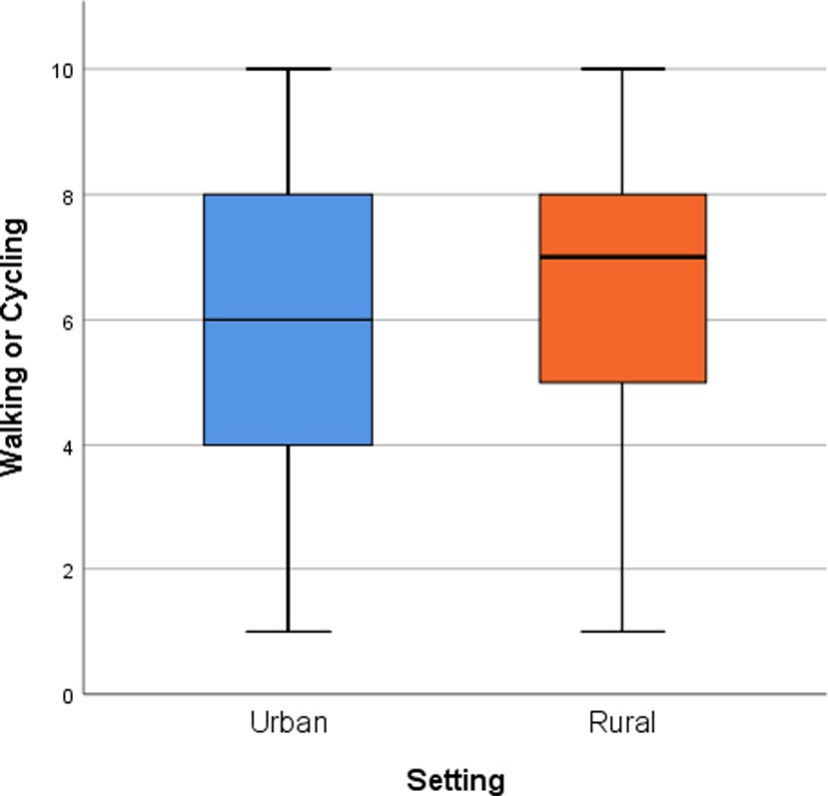	**Common results:** Perception of having sufficient basic services. Local businesses that allow for the purchase of essential products. Demand for improved healthcare services and more public services.
	**Urban context:** Presence of public administration offices. Wide variety and availability of businesses. Demand for daycare services.
	**Rural context:** It is necessary to travel to another municipality to access supermarkets and the secondary school. Prices of essential products are higher in local stores. There is a perceived need for more restaurant options in the municipality.
	**Interpretation**: Despite the existing variations between the two environments regarding the commercial offer, diversity of services, and specific demands by population groups, overall satisfaction in both contexts does not differ significantly. However, in the rural setting, there is greater variability in perceptions related to generational differences. Older individuals tend to view local businesses and existing public services more positively, while younger individuals are more critical, expressing the need for more shopping options and an improvement in the available public services.
*p-value > 0.05* 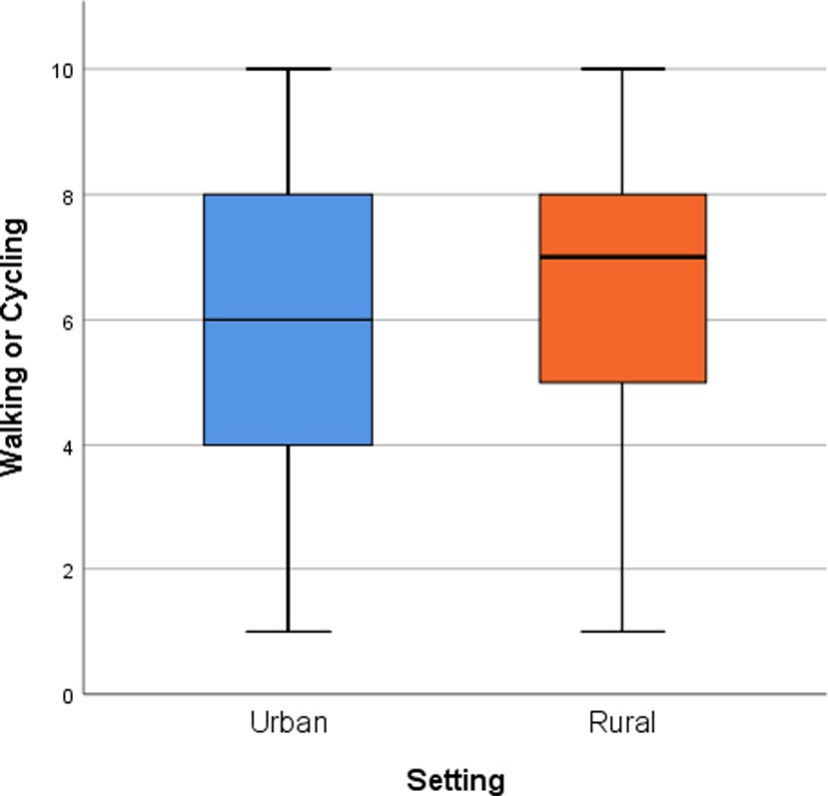	**Common results:** Limited employment opportunities, which encourage migration to other places in search of better job prospects. Potential areas for the development of new local businesses that generate employment for the local population was identified.
	**Urban context**: Employment is precarious and seasonal, influenced by the tourism sector. The young population and those older than 45 face the greatest difficulties in accessing the job market. Migration is common to seek skilled employment opportunities. Challenges are identified in establishing and maintaining local businesses. There is a strong economic dependency on tourism that shapes the local job market.
	**Rural context:** Lack of local businesses and industrial estate development. Migration is common to seek employment. Identified potential areas for local development include catering, tourism, and the environment.
	**Interpretation**: There are common challenges and issues in terms of employment, such as precarious jobs, a lack of opportunities, and the need to develop alternative economic sectors. Despite these differences, the perception of work and the economy in both municipalities shows similarities. It is noteworthy that the tourism sector is perceived as both a potential opportunity and a challenge for local development. The variability of opinions in the rural context is related to better expectations among younger individuals with higher levels of education.
*p-value < 0.05* 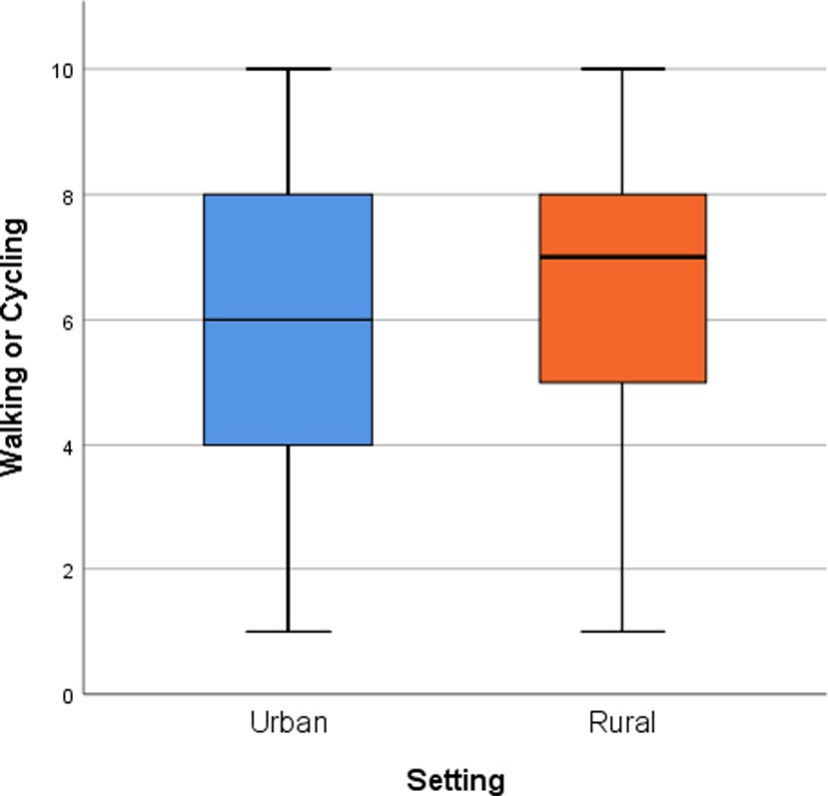	**Common results:** Perception of a shortage of housing in suitable conditions and high prices. Difficulties in accessing housing for young people and individuals with fewer resources. Lack of local policies that promote access to housing. Emigration to nearby municipalities due to a lack of housing.
	**Urban context:** Tourism is pointed out as the main cause of the lack of long-term rental housing and rising prices.
	**Rural context**: Limited availability of housing in the market is perceived. Homeownership predominates, with a significant number of vacant homes. The presence of second homes is noticeable.
	**Interpretation**: The price of housing, difficulties in accessing it, and the higher cost of living in the urban setting adversely affect the perception of this theme. Older individuals give higher ratings in the urban context and lower ratings in the rural context. The underlying reasons point to architectural barriers and the economic cost of maintaining large single-family homes as determining factors. Both contexts exemplify current housing issues in Spain, such as rising prices due to strong tourist demand and depopulation in rural areas.
*p-value < 0.05* 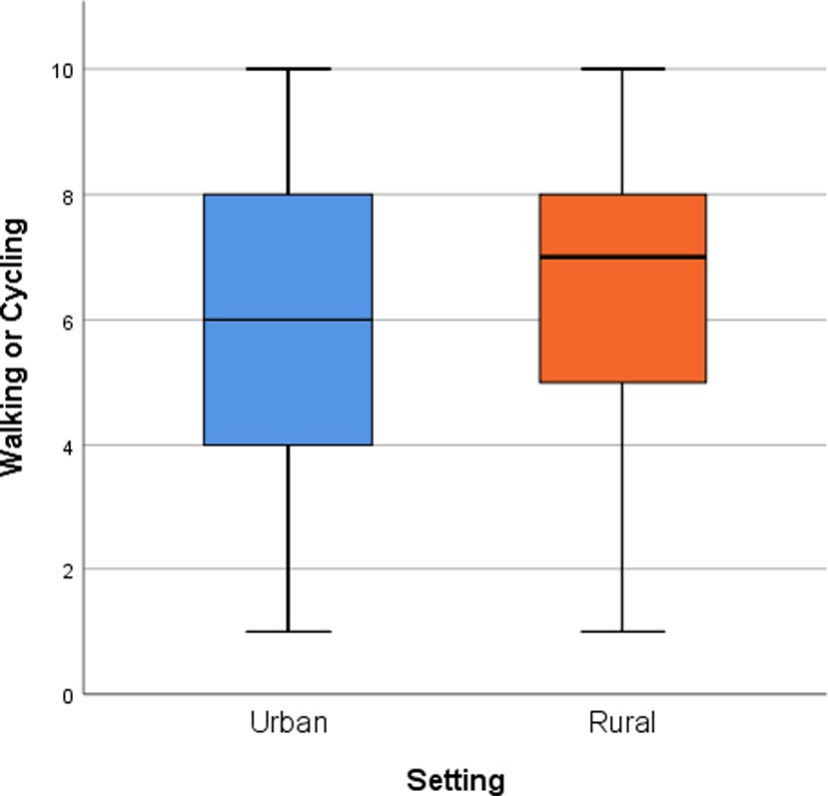	**Common results:** Associative fabric is considered essential for social interaction. The existence of associations is valued as spaces where people can meet and establish social relationships.
	**Urban context:** Spaces and activities for adults are identified. There is a demand for free activities. Language is perceived as a barrier to social contact. Loneliness issues are noted in older people and foreigners.
	**Rural context: Demand** for spaces and activities for children and adolescents. Demand for activities for the older adult/adults and during winter and weekends. The village is considered a space for social contact.
	**Interpretation:** Closer and more intimate social interactions leading to greater community cohesion occur in the rural area, thanks to its dense network of associations, compared to the greater cultural diversity and availability of public spaces in the urban area. The rural municipality benefits from a robust network of associations and a less dense population, which promotes greater social interaction. In the urban context, activities and spaces are more structured, although they present accessibility challenges for all community groups. Meanwhile, the low population density in the rural area hinders the implementation of a wide and varied range of activities.
*p-value < 0.05* 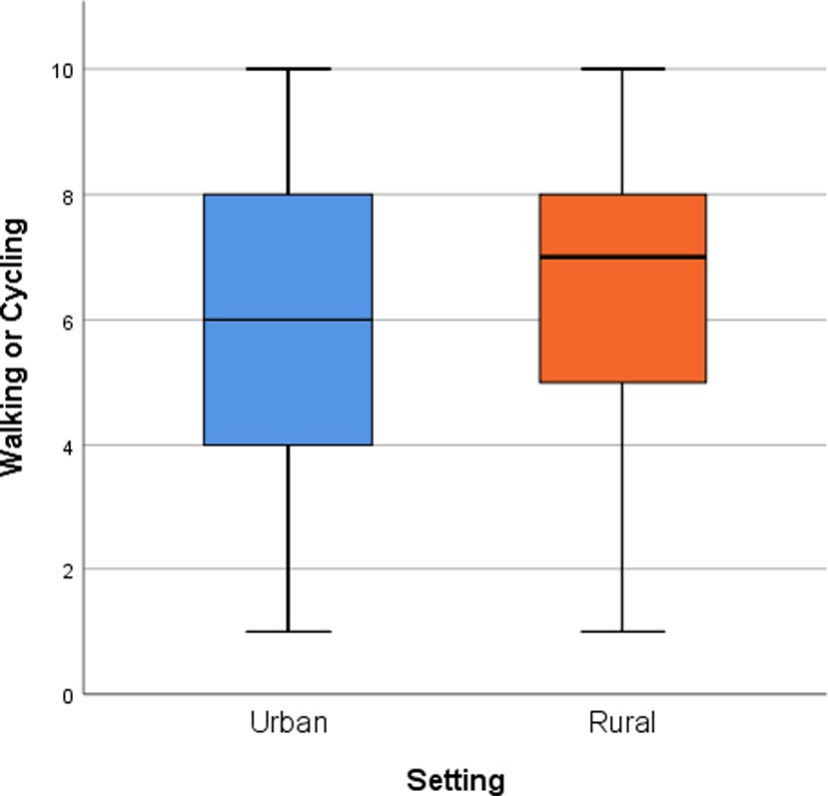	**Common results:** In both contexts, there is a strong sense of identity and belonging rooted in their community.
	**Urban context:** The natural environment, community openness, and cultural diversity are factors that promote positive identity and belonging. The lack of integration of non-EU foreign individuals is a limiting factor for belonging.
	**Rural context:** Strong sense of identity related to the associative network and belonging to a small community. Difficulties in integrating new people into the municipality.
	**Interpretation:** The greater presence of migrants and cultural diversity has a more pronounced impact in the city, resulting in a less favorable evaluation of this topic. In the rural context, where the community tends to be more cohesive, a stronger sense of identity is promoted. Differences are linked to the reasons people have for residing in the municipality. Those who were born in the place and own property tend to experience a greater sense of belonging, while those who migrated for work-related reasons and have temporary residence may feel a lower degree of identity and connection.
*p-value > 0.05* 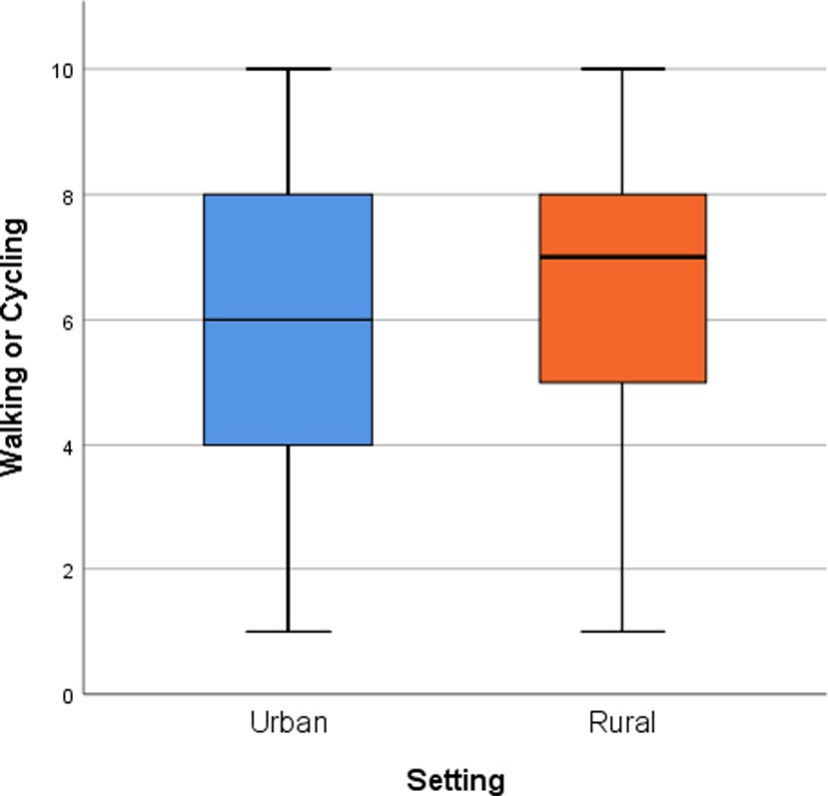	**Common results:** There is a positive perception of the sense of security. Concerns about safety are addressed in terms of road safety, streets and public space lighting, and police presence. Insecurity is related to home burglaries, vandalism, and behaviors associated with alcohol consumption in public spaces.
	**Urban context:** There is a perceived lack of police presence during summertime, which is the peak tourist season with more nighttime leisure activities. Women feel unsafe when walking at night in certain areas of the city.
	**Rural context:** Few incidents are reported. The presence of the police is seen with mixed feelings.
	**Interpretation**: In both contexts, safety is a shared concern, especially regarding the presence of the police and the feeling of insecurity at night and in specific areas. The lack of local police is the factor that most influences the variability of opinions in the rural context. On the other hand, in the city, the diversity of opinions is more related to the location of the residence, areas with isolated houses and those with nightlife venues being perceived as less safe.
*p-value > 0.05* 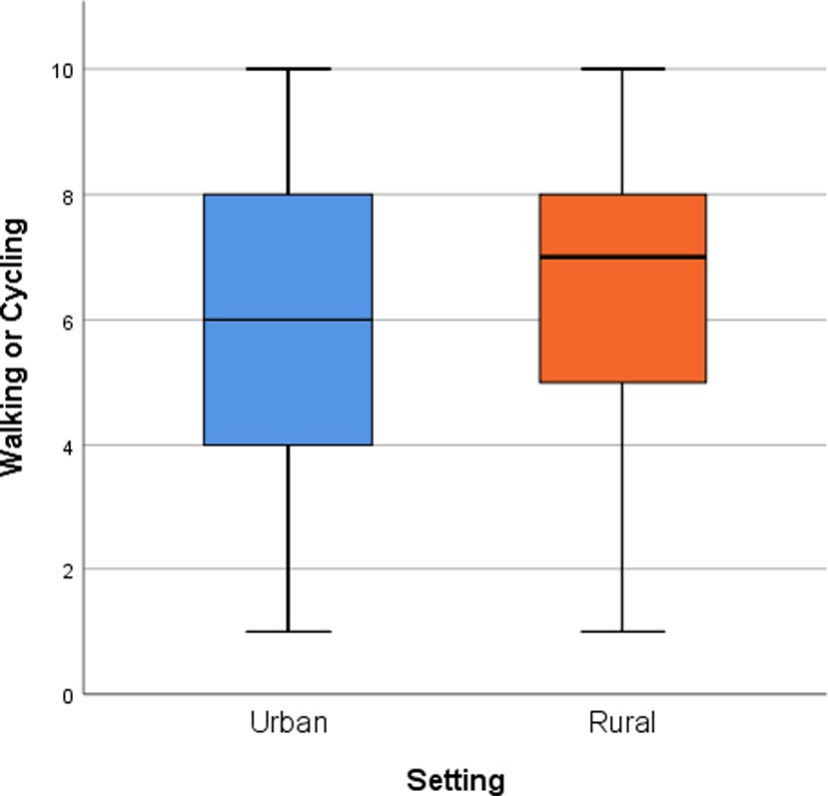	**Common results:** The common factors are the lack of care and maintenance of natural spaces and the uncivil behavior of the citizens as a source of cleanliness problems. Proper use of garbage and recycling bins is not being practiced.
	**Urban context:** Municipal cleaning efforts in urban spaces are positively evaluated, but negatively in natural areas. Some neighborhoods are perceived as cleaner than others.
	**Rural context:** Recognition of the municipal administration’s efforts in care and maintenance.
	**Interpretation**: The common perception revolves around the role of the community and the local administration in caring for and maintaining the environment. In both contexts, citizens are seen as responsible for the cleanliness of spaces and the improper use of waste disposal systems. Additionally, the work of local administrations is recognized, though more positively in the rural setting. In the urban context, opinions vary depending on the area of the city where one lives, commercial areas and the city center receiving higher evaluations.
*p-value > 0.05* 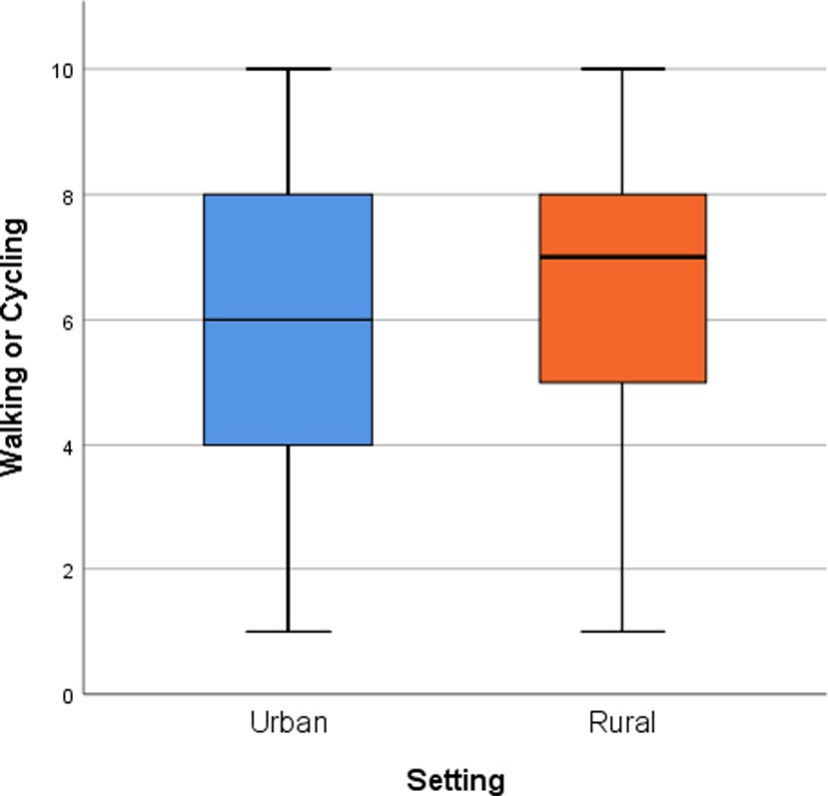	**Common results:** There is confusion between participation and informational and consultative channels. Associationism and social networks are identified as effective channels of participation. The lack of a participation culture is explained by the lack of involvement, attendance in processes, and interest in monitoring actions, both on the part of citizens and the local government.
	**Urban context**: There is a wide variety of participation channels. Lack of changes or improvements despite citizen participation.
	**Rural context:** Participation channels are related to accessibility to the local government. There is more informal participation outside the established channels. Lack of listening processes by the local government.
	**Interpretation**: The variety of participation channels, accessibility to local authorities, and transparency in decision-making are factors related to a better perception of this dimension. Associative networks are identified as a collective force that promotes listening rather than actual participation in processes. Lower scores in the urban context are associated with a limited perception of participation in decision-making and process transparency. In the rural context, this is linked to the presence of few organized and structured collective participation processes and limited community involvement in matters related to their municipality.

## 4 Discussion

### 4.1 Main findings

In this study, the performance of the EdV tool is described in a real-world context through its pilot testing in two municipalities. Its testing in both rural and urban settings using a heterogeneous sample of population groups has provided results that support the idea that the EdV tool allows for a better understanding of the perception of the impact of the physical, social, and economic environment on the community and how this impact manifests in reality. The findings reveal differences in the perception of places between residents of urban and rural municipalities, with an overall more favorable assessment of the rural municipality. Overall, *Public Transport* and *Work and Local Economy* are the areas rated the lowest by all social groups and contexts, *while Identity and Belonging* is the best-rated dimension (Table 4).

The differences in perception between the rural and urban contexts are significant in the entire mobility block (*Walking or Cycling, Public Transport, Traffic and Parkin*g), spaces (*Natural Spaces* and *Streets and Spaces*), *Housing and Community, Social Interaction, and Identity and Belonging*. We believe that these differences are due to the perception of mobility experienced in each evaluated context.

Similar results were obtained in previous studies (7–12, 56, 71), in which active mobility is influenced by the condition of streets and sidewalks, the perception of safety, architectural barriers, affecting both people with reduced mobility and care-related activities, the aesthetics of streets and spaces, and the availability of amenities, such as benches, public toilets, trees, and shaded areas. The lack of shade, noted as one of the determining factors in the use of spaces, is contextualized in the Mediterranean climate of this region, which has long, hot summers and increasingly frequent and prolonged heatwaves. This aspect was consistent with studies exploring the relationship between the area and the mobility of older people (10, 38).

Despite having favorable topography and climate, cycling mobility is not fully developed due to the absence of specific infrastructure and promotion of its use. While in the urban context, it is considered important to promote active transportation within the city, in the rural setting, it is more related to recreational and intermunicipal activity. In both contexts, and similarly to previous research (4, 10, 13, 14), the main barrier identified was the perception of insecurity, associated with the organization of the public space, where car dominance prevails, and the lack of a well-connected network of bike lanes, and signposted cycling routes separated from road traffic.

It is noteworthy that, while *Public Transport* received the most negative ratings in both contexts, the distinguishing discourses revolve around deficient connections, intraurban in the city and interurban in the rural area, and between essential services and amenities (hospital, high school, shopping areas). In cities, active transportation (walking, cycling, and use of public transport) is associated with health benefits due to increased physical activity and reduced exposure to environmental pollution (4, 16, 17), where having various means of public transportation, nearby stops connected to each other and financially accessible, are factors that promote physical activity and reduce the use of private vehicles (4, 5, 18, 38). In rural areas, with a widespread negative evaluation, as seen in other studies (38, 55, 56), differentiated approaches are required to promote active transportation and connected communities (43), where the older adult/adults population has lower mobility and autonomy to drive their own vehicles (12, 39, 40). Transportation policies and planning influence active mobility, private vehicle use, transportation options, air quality, and noise levels in municipalities (5, 15, 30). The lack of public transport or barriers to its use, especially in rural areas, exacerbates health inequalities related to access to healthcare resources and social exclusion (5, 17, 39, 72).

In the urban context, traffic was identified as a deterrent to active transportation, both due to the insecurity it generates for pedestrians and cyclists, and the air pollution and noise it produces. The negative impact of traffic has been documented in numerous studies (5, 10, 38, 55, 56, 71), showing a direct correlation between the negative perception of traffic and population density, with a more favorable assessment in rural areas.

The evaluation of *Natural Spaces* showed significant differences between the two contexts, despite being assessed very positively in both municipalities. The urban community viewed favorably the presence of accessible, well-maintained parks and beaches, while the rural community highlighted the natural spaces located around the municipality. Current evidence suggests that green and blue spaces promote health by encouraging physical activity, emotional well-being, and social interaction, and mitigating the impact of heat, noise, and pollution (5, 19, 20, 35), thus reducing cardiovascular morbidity and mortality and cardiovascular risk factors, such as obesity, stress, high blood pressure, and type 2 diabetes (20, 32). Additional findings indicate that, in the perception of natural spaces, quality is more relevant than the total quantity of natural areas available in the setting (20).

In the urban evaluation of *Housing and Community*, the difficulties in acquiring housing, both for purchase and rent, were mainly attributed to tourism. In this regard, vulnerable individuals face more challenges in accessing housing, with unequal distribution based on material resources. Those with lower financial capacity experience greater housing instability, access lower-quality housing stock, and reside in more disadvantaged neighborhoods with poorer access conditions (5, 33, 73). In Spain, over recent decades, national and regional policies have promoted an oversized and underutilized housing stock with ownership as the primary access route, leaving renting as the last resort for individuals without resources and without a social housing stock to provide a stable solution for those facing greater difficulties (33). The problems resulting from the touristification of urban areas primarily impact rentals by increasing prices and worsening rental conditions, thereby preventing these groups from accessing suitable housing (33, 74). On the other hand, in the rural context of the evaluation, there was a lack of agreement with previous studies that associated the impact of housing on health with factors, such as architectural barriers for older adults, isolation, total surface area, and housing maintenance (5, 38, 75). In contrast, the findings of this study suggest that the effects of tourism and depopulation as factors hindering housing accessibility are perceived as more important than the physical characteristics of the housing itself (5, 75–79).

Both the discussion groups and interviews conducted in the urban area highlighted tourism as a major influencing factor in areas such as transportation, traffic, housing, employment, and cost of living. The impact of tourism has been addressed in previous research, which has identified it as a driver of rising housing prices, increased traffic and noise, and precarious seasonal employment conditions in cities (80–82). Moreover, dependence on tourism can lead to economic vulnerability, as evidenced during the COVID-19 pandemic (80, 81, 83).

Similar to other studies (71, 83), the sense of identity and belonging was higher in rural areas than in urban ones. It was inversely proportional to the size of the community and increased with homeownership, participation in community activities, length of residence in the municipality, and being born in or choosing it as a place to settle. In the urban context, it was found that foreign-born individuals, especially Africans and Latin Americans, expressed a lower sense of identity and belonging due to integration difficulties and limited participation in local culture-related activities, such as festivals and the local language (55, 71, 83, 84).

Previous research (38, 55, 71) has highlighted the importance of social contact and its relationship with emotional well-being and identity and belonging (56, 85). This relationship is particularly relevant in older adults, where the perception of the environment has a greater impact than their social status (82). In rural contexts, it has been observed that social interaction, especially during childhood and old age, benefits from the availability of essential services, such as shops and public transportation, as well as meeting spaces, like squares, community centers or well-maintained parks (21, 22, 39, 86). When these spaces are energized with activities that promote social contact, greater interaction among people is fostered. This is essential as the use of certain spaces is influenced by the degree of social interaction they facilitate. Furthermore, neighborhoods or municipalities with high social cohesion and community participation are capable of mitigating the impact of poverty during adolescence (87).

Social contact plays a fundamental role in reducing the perception of loneliness. Loneliness decreases as social interaction increases, participation in group activities, access to basic facilities, and having access to public transport (34, 88–92). Scientific literature presents divergent views on how the rural or urban areas impacts the experience of loneliness. In this context, it is noteworthy that subjective assessment of the quality of the setting has a greater influence than the objective characteristics of the environment itself (34, 41, 42, 93).

### 4.2 Utilization of the PST and performance in real environments

The PST has been used in numerous countries and contexts (94), and it is widely implemented in Scotland (55). Its main objective is to facilitate community participation in the development of local plans, assess the quality and characteristics of specific municipalities or neighborhoods, such as Skopje, Nicosia, and the municipalities evaluated in this study (55, 56, 71), by the general population or specific population groups (38). It has also been used in the evaluation of specific dimensions, such as natural spaces or housing (95, 96), or as a basis for the development of other environmental analyses (72).

Previous studies have documented difficulties regarding the approach used for territorial delineation (38, 55, 72). The administrative demarcation of an area, whether it’s a neighborhood or a municipality, can lead to a portion of the community not feeling involved in the evaluation process. This issue was evident in the context of the Identity and Belonging theme. This problem can manifest in both neighborhoods and rural districts that are administratively associated with a municipality but have their own identity, as seen in our study with the rural district of La Xara in Denia. The determination of the territorial scope subject to evaluation should align with the inherent purpose of the evaluation, so that the results obtained are relevant in that territory (3). Additionally, in neighborhood evaluations, the perception of this dimension can vary between being considered at the neighborhood level or at the city level, depending on the interviewed person. Therefore, it is of utmost importance to define the territory to be evaluated appropriately, incorporating criteria beyond purely administrative aspects.

Similarly, to the Scottish experiences (55), involvement of the local government and municipal technicians in designing the participation strategy favored the inclusion and representativeness of the participating population in the study. The online option was the least effective in our case, especially in the rural setting, whereas discussion groups and in-depth interviews provided the most comprehensive and profound results. The group methodology was the most advantageous because it allowed us to understand the unequal experiences of individuals in relation to their municipality and motivated them to participate in the subsequent phases of the process. The project’s goal required a higher level of effective participation rather than a large number of participants. However, despite the meticulous conception of participatory processes, and similar to the experiences observed in Scotland, Macedonia, and Cyprus (55, 56, 71), a pattern of greater female participation was evident at an approximate rate of 63%, while the average age ranged from 42 to 57 years (urban and rural populations, respectively).

In our case, aligning the evaluation of places with the XarxaSalut Strategy (61), which already incorporates participation structures, improves the implementation of the tool. These intersectoral platforms, including local government, citizens, and technical personnel, facilitate the design of evaluation processes and the development of actions stemming from PST results aimed at improving the health of the population with an equity focus. On the other hand, despite having a Network of Health-Promoting Municipalities (XarxaSalut) since 2017 that requires community participation in the development of all its actions, the inclusion of participation in joint decision-making is still incipient. Unlike Scotland (97), there is no specific legislation that obliges community involvement in policy formulation and service delivery (98).

Regarding its use, it is a lengthy tool, where the collection of qualitative information, its organization, and analysis require resources and technical expertise. To guarantee a proper implementation of the tool, the process must be carried out by the local government or have specific funding.

The organization of the 14 areas to be evaluated presents challenges described in previous experiences (55, 72), such as the repetition of topics (*Feeling Safe* or *Care and Maintenance* in *Walking or Cycling*, *Streets and Spaces* or *Natural Spaces*) or the inclusion of two topics in one theme (*Traffic and Parking* or *Walking and Cycling*). In our study, we also detected the difficulty of assessing aspects that are not known (such as *Public Transport* if one is not a user) or that certain areas, depending on the evaluated territory, work at the city level and not at the neighborhood level (e.g., *Work and Local Economy*). On the other hand, its design with open-ended questions and overlapping topics allows for the inclusion of aspects that are not specifically asked about (e.g., an urban vacant lot as a space that promotes social contact) and highlights the challenges and opportunities of the context for people in more vulnerable situations.

One of the strengths of the tool is its ability to identify the wealth and strengths of neighborhoods and municipalities, beyond the deficits they may have. When used with a salutogenic approach, it has the potential to show the dynamics of people’s interaction with their environment, the opportunities it offers, and how challenges are confronted. The PST allows for the incorporation of the Sense of Coherence into places, helping to understand what people perceive and experience in their surroundings in terms of comprehensibility, manageability, and meaningfulness (99).

As in previous experiences, the challenge of managing expectations generated during the evaluation process has been observed (12, 55, 71). Community participation may be compromised in the absence of feedback on the results, the lack of implementation of actions derived from the process, and transparency in decision-making (5, 26, 45, 51, 96, 97, 100). To avoid this threat, it is essential to define the purpose of participation in advance, i.e., whether it is about collecting information or initiating a joint decision-action process (50, 55). In any case, both the role and responsibility of those conducting the evaluation process and the individuals or institutions responsible for carrying out the prioritized actions should be described (50, 55).

Given the above, it is advisable to incorporate PST as part of a process rather than considering it an end in itself, where the tool represents another step-in community participation rather than just a citizen consultation. It is desirable to orient the tool toward action, where the feedback of results is linked to the proposal, prioritization, and implementation of actions within a continuous community process and promotes interdisciplinary technical work based on prioritized actions. Results alone do not generate substantive improvements, and their impact will depend on the available resources and the commitment of both citizens and local administrations (51, 55). The existence of previous community participation experiences at the local level facilitates the development of processes to improve places (55, 56, 71).

Having validated and effective participatory tools for diagnosing deficits and assets in environments makes it easier to involve the community in decision-making about the design of neighborhoods and towns and connects institutional processes with the needs of citizens. Furthermore, this allows for the incorporation of equity as long as the process addresses different axes of inequality and incorporates a “territorial” perspective into the processes (98). It is important to note that lower levels of involvement in participation (information and consultation) do not have a significant impact on transforming the balance of power between citizens and local governments and people’s living conditions (101).

### 4.3 Strengths and limitations

The main strength of this study is the use of mixed methods, which has allowed for a deeper understanding of how the physical, social, and economic context impacted communities and the differentiated experience between rural and urban settings. The incorporation of both quantitative and qualitative approaches in the research design increases the reliability of the results, enabling a solid understanding of the perception of their environments in two different populations. Despite not having a representative sample, as heterogeneous participation was prioritized over a large number of participants, the high participation rate in the study is noteworthy, facilitated by the implementation of three different data collection techniques. The diversity of participants and the richness of their discourses and perceptions have provided information about the facilitating elements and potential barriers in rural and urban contexts, which can be generalized to other municipalities with similar characteristics.

Another strength of the study is the use of the PST within the framework of the XarxaSalut strategy, with the support of the local government, technical staff, and associated citizens. This synergy has made it possible to align the research objectives with community action processes (61) that were being carried out in the municipalities of Denia and Yátova at the time of the study.

Regarding the limitations of the study, despite seeking broad community participation, the pattern of greater female participation and representativeness in terms of age may have influenced the results. Moreover, the data analysis did not differentiate between modes of participation, solely categorizing them by urban or rural context. This approach prevented the identification of potential variations in municipalities’ perception based on participation modes, limiting a more nuanced understanding of factors influencing diverse population groups. Another limitation arises from the absence of a specific sample size, potentially impeding the generalizability of findings and compromising precision in detecting significant differences between contexts. Additionally, this limitation restricts the applicability of results to other regions with different sociodemographic contexts due to their specific territorial nature. Additionally, the implementation of the PST can be demanding in terms of time and resources, which could limit its applicability in other localities without adequate support. Nevertheless, this study lays groundwork for exploring disparities between urban and rural areas concerning environmental perception. It underscores the imperative for future research endeavors to employ larger, more representative samples to achieve a more comprehensive understanding of these dynamics.

It is important to mention that the completion of this study coincided with the onset of the COVID-19 pandemic, which made it impossible to provide adequate feedback to the community. On the other hand, its development in pre-pandemic phases means that it cannot take into account possible changes in residents’ perceptions of relevant issues during and after the pandemic, such as the digital divide, housing characteristics, access to healthcare resources, availability of outdoor activity spaces, and community networks. Furthermore, it should be taken into consideration that the recent intensification of the effects of climate change in Spain may require the incorporation of a climate perspective into the tool, as is being developed in Scotland or Germany (102, 103), to assess its impact in the context of neighborhoods or municipalities.

It is essential to use subjective approaches to understand the influence of environments on different social strata and how individual conditions can impact these perceptions. Future research should address these issues, as well as compare differences in participation methods, exploring possible disparities and nuances in the results obtained.

## 5 Conclusion

In this study, we have verified the capacity of the PST to deeply understand the communities’ experiences regarding their contexts. The examination of its performance in both rural and urban settings, utilizing a heterogeneous sample encompassing various population groups, has yielded outcomes substantiating the notion that the PST facilitates a comprehensive understanding of community perceptions regarding the physical, social, and economic environment and their tangible manifestations. Notably, disparities in place perception have emerged between residents of urban and rural municipalities, with the rural setting generally being more favorably rated. Remarkably, Public Transport and Work, as well as Local Economy, emerge as the dimensions garnering the lowest ratings across all social groups and contexts, whereas Identity and Belonging emerges as the most highly rated dimension.

The results support that PST is a valuable tool for promoting local health due to its versatility and action-oriented approach. It introduces a structure and methodology that allows for discussions in terms of social determinants and identifies how the place where people live conditions health. Its implementation facilitates the starting point for developing prioritized, intersectoral, and participatory local actions aimed at addressing health inequalities.

## Data availability statement

The raw data supporting the conclusions of this article will be made available by the authors, without undue reservation.

## Ethics statement

The study was conducted in accordance with the Declaration of Helsinki and approved by the Institutional Review Board (or Ethics Committee) of the University of Valencia (Spain) (1208176 data of approval 08 November 2019).

## Author contributions

AO-O: Conceptualization, Data curation, Formal analysis, Investigation, Methodology, Project administration, Supervision, Visualization, Writing – original draft, Writing – review & editing. VG-C: Conceptualization, Methodology, Writing – review & editing. RJ-V: Data curation, Formal analysis, Methodology, Writing – review & editing. RP: Conceptualization, Funding acquisition, Investigation, Methodology, Project administration, Resources, Writing – review & editing. EP-S: Conceptualization, Funding acquisition, Investigation, Project administration, Resources, Writing – review & editing. IS-A: Data curation, Formal analysis, Writing – review & editing. TS-S: Data curation, Formal analysis, Writing – review & editing. MG-C: Data curation, Formal analysis, Writing – review & editing. JP-C: Conceptualization, Investigation, Methodology, Visualization, Writing – review & editing.
