# Supplementary material for: Health equity in urban and rural settings: implementation of the place standard tool in Spain
Source: Front Public Health. 2024 May 9;12:1292032. doi: 10.3389/fpubh.2024.1292032 (PMC11129683; doi:10.3389/fpubh.2024.1292032)
Supplement: Supplementary file 1 [file Presentation_1.pdf]

frontiers

Supplementary Material

Health equity in urban and rural settings: implementation of the Place Standard Tool in Spain

1 Supplementary Figures and Tables

1.1 Supplementary Figures

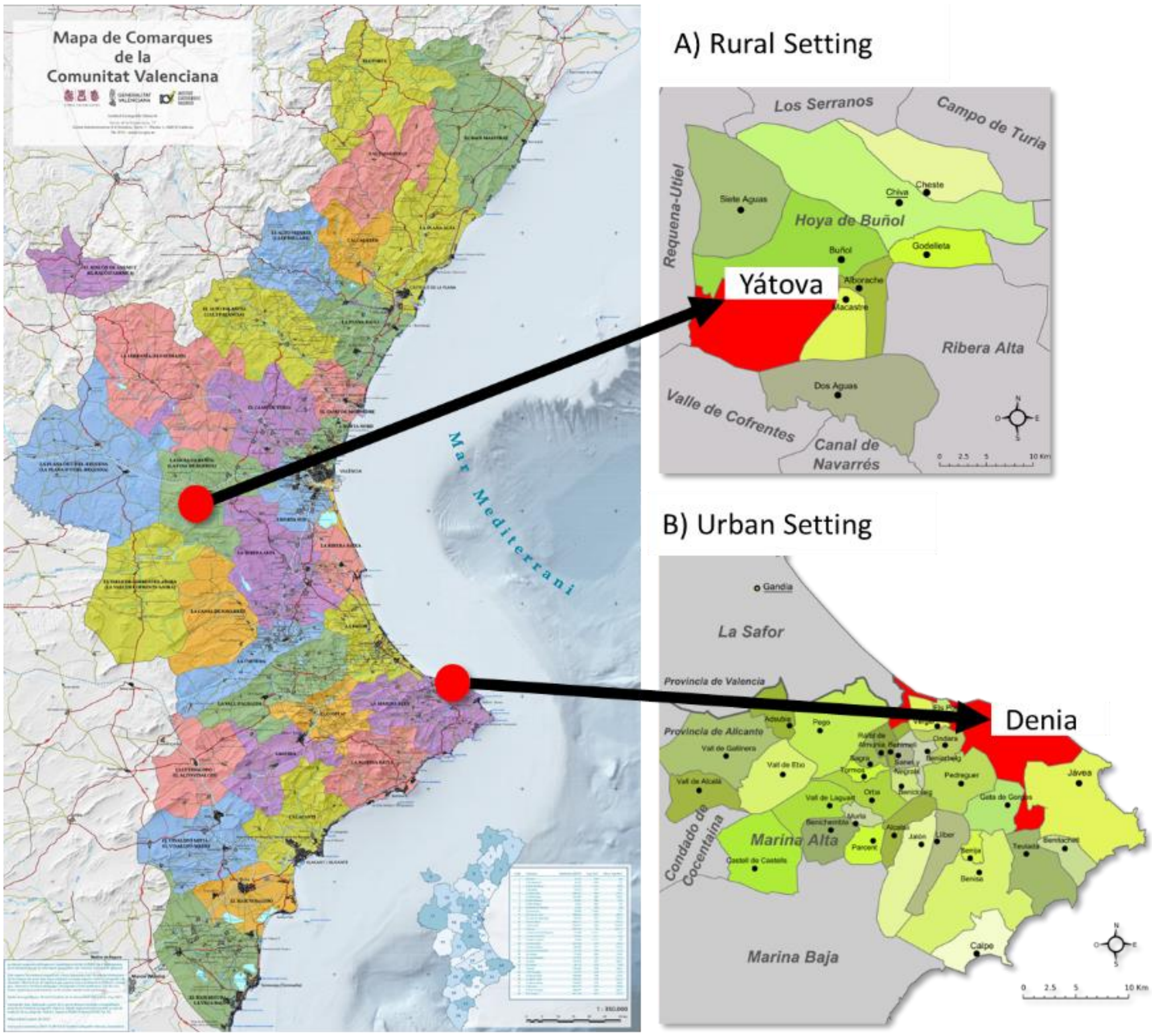

Image: Institut Cartogràfic Valencià. Conselleria de Medio Ambiente, Agua, Infraestructuras y Territorio. Generalitat Valenciana.

Supplementary Figure 1. Studied municipalities in the Valencian Community (Spain). Municipality A) represents the rural context, and B) represents the urban context

1.2 Supplementary Tables

Supplementary Table 2. Description of the municipalities under study

Characteristics	Urban setting	Rural setting
Surface area (km^2)	66.18	120.24
Population density (hab/km^2)	649.04	17.56
Altitude (m)	16	435
Geography	Coastal Municipality	Inland Municipality
Distance from the capital (km)	100	46
Total population	42,953	2,112
Aging[1] (%)	134.3%	195.6%
Population–Spain (%)	77.4	95.7
Population–EU (%)	8.9	2.0
Population–Other countries (%)	13.7	2.3
Disposable income per tax return (€)	20,214	18,690
Active companies by sector of activity (%)	80.88% Services[2]	77.67% Services[2]
	15.60% Construction	18.75% Construction
	3.42% Industry	3.57% Industry
Municipal budget (€)	70.798,427	3.180,009

[1]Ageing Calculation: (Pop.>64)/(Pop.<16)×100

[2]The services sector consists of commerce, hospitality, and transportation.

Supplementary Table 2. Qualitative results: English Translation of Verbatim

PARTICIPANTS' EXPERIENCES IN RURAL AND URBAN CONTEXTS	
**1. WALKING OR CYCLING**	
**Urban context**	
V1	You can walk everywhere here. (GD5)
V2	There's an avenue where seniors walk, and there isn't a single bench. (GD8)
V3	There's a lack of trees on the streets to provide shade and cool down the environment in the summer […] there are streets where you can't escape the heat. (GD8)
V4	Public toilets are needed on the road to Les Rotes (beach promenade). (GD8)
V5	There are benches, streetlights, and signs that make it difficult to walk on sidewalks for wheelchair users, the blind, parents with strollers, and people carrying groceries. (GD2)
V6	In general, bike lanes are not safe; they are full of potholes and very narrow. Often, you have to ride on the sidewalk because the bike lane suddenly ends […] and you have to go on the road to the beach with cars passing too close. (GD7)
V7	Crosswalks are not respected; cars don't stop. (GD3)
V8	The area of Les Marines is dangerous for walking; you have to go on the shoulder, and there's heavy traffic. (GD2)
V9	There's always traffic in front of the school, and it's dangerous. (GD2)
V10	There are places where you feel scared when riding a bicycle; cars don't respect cyclists. (GD5)
**Rural context**	
V11	You can walk everywhere; this is a small town. (GD2)
V12	There are many streets with very narrow, sloping sidewalks, full of potholes […] you can't use a wheelchair or a walker, there are no ramps anywhere, or lowered curbs, you have to walk in the middle of the street. (GD3)
V13	There are many hills, and not everyone can go up to the supermarket located in the upper part of the town. . (GD8)
V14	There are places that don't even have a bench to sit on. . (GD8)
V15	If you ride a bike, you have to use the road with the cars; there's no other way. (GD5)
**2. PUBLIC TRANSPORT**	
**Urban context**	
V16	Even the hospitals are poorly connected by bus […] if you can afford it, you take a taxi, otherwise, you lose the whole day. (GD5)
V17	It's impossible to go to neighboring towns like El Verger or Pedreguer; everything starts and ends in Denia. (GD4)
V18	You have to drive the children to school; there is no bus. (GD2)
V19	The shopping center is very poorly connected, and if, for example, you work there or go to the movies and finish late, you have no way to get back because the last bus leaves early. (GD7)
V20	I have to walk to pick up my daughters from school because I can't afford three one-way tickets and three return tickets every day. (GD2)
**Rural context**	
V21	If you don't have a car, you are disconnected. (GD2)

V22	Not all buses have wheelchair ramps; you have to call ahead, or it’s a lottery. (GD3)
V23	The train and bus schedules are not coordinated; either you arrive long before the train comes, or you have to wait a long time[…] Same for the return trip; in the end, you lose the whole day. (GD5)
V24	There is no good connection to go to the Manises hospital; you have to drive there. (GD8)
V25	The last bus from the train station leaves at 7:20 p.m., and it’s impossible to work in shifts or at night in other towns. (GD2)
V26	This is a problem throughout the region. (GD1)
**3. TRAFFIC AND PARKING**	
**Urban context**	
V27	People want to park in front of their homes, and that’s not possible; there’s not enough space for everyone. (GD6)
V28	There’s not enough space for all the cars. (GD5)
V29	There are avenues where there’s a lot of noise and pollution because lots of trucks pass by […] During summertime, you can’t leave your windows open. We would need a bypass road to divert them outside the city. . (GD6)
V30	[…] people use the car for everything; there are trips that would be faster on foot. (GD4)
V31	There are traffic jams every day at the school entrance. (GD2)
V32	During summertime, the traffic is unbearable. (GD5)
**Rural context**	
V33	You can park almost anywhere. (GD2)
V34	During summertime, traffic rules and parking regulations aren’t followed, but as the population multiplies, it becomes more permissive. (GD1)
V35	There’s hardly any traffic. (GD5)
V36	People use their car to go to places that are just as close to walk to […] even to take their kids to school, even though it’s nearby. (GD8)
V37	The healthy habit of walking is being lost. (GD8)
**4. STREETS AND SPACES**	
**Urban context**	
V38	Depending on where you go, there are areas with benches and shade, or not. (GD8)
V39	During summertime, there are streets where you can’t walk because of the heat. Trees should be planted to provide shade and cool down the environment a bit. (GD5)
V40	It’s very nice to walk in the city center […] the castle is the city’s treasure. (GD4)
V41	Lighting should be improved in some streets that have dark corners […] and there should be an order in the city’s aesthetics; in some streets, every building looks different. (GD6)
**Rural context**	
V42	It’s very pleasant to walk around the village; it’s very beautiful and peaceful. (GD2)
V43	In the town square, there’s a steep slope; it would be good to have a railing. (GD8)
V44	During the winter, there are some streets of the upper part of the village that have no lighting. (GD2)
V45	More trees in the streets would be nice to give them a different look. (GD5)
**5. NATURAL SPACES**	
**Urban context**	

V46	Natural spaces have improved a lot in recent years; we have incredible beaches, mountains nearby, a natural park, a greenway, and a promenade by the sea… (GD5)
V47	Accessibility to natural spaces has improved a lot […] there's even a beach adapted for people with disabilities. (GD1)
V48	All the cleaning resources go to the beaches due to the algae problem. (GD6)
V49	We need more parks in the city center and Las Marinas (beach area). There are only five in total for the whole city. (GD5)
**Rural context**	
V50	The city council has worked hard to take care of the natural environment, and they have made significant improvements in the Cueva de Las Palomas […] there are projects to conserve the environment and policies aimed at preserving it. (GD1)
V51	The area around Mijares need some enhancements. (GD1)
V52	We are surrounded by a beautiful mountain range. (GD8)
V53	San Vicente Park is fantastic. (GD2)
**6. PLAY AND RECREATION**	
**Urban context**	
V54	We lack more cultural activities, such as theater and concerts, and the cultural center closes on Sundays, which is when you can go out. (GD5)
V55	We lack a theater or an auditorium. (GD6)
V56	The sports center is in poor condition […] you can't do sports there without paying. (GD5)
V57	The schools are closed on weekends, and children can't play where there are courts and soccer fields. (GD5)
V58	There is nothing for us apart from bars, pubs, and the nightclub; you can walk up and down the street. (GD7)
V59	Entertainment is expensive; you have to pay for everything. (GD4)
V60	This center (Llunàtics youth center) is very good; it's the best in the region. Here, we can decide what activities to do; it's a meeting place. We feel like we contribute, and it belongs a bit to us. (GD8)
**Rural context**	
V61	There are several sports facilities; we have a gym, a fronton court, soccer and basketball fields … there are Pilates, Zumba, and Karate classes … and there are groups of people who go out for walks. (GD1)
V62	In the senior center, you pay a fee and only play bingo; there's nothing else … if bingos were organized every day, it would always be full. (ES8)
V63	There's nothing for older people, and you end up in front of the TV all day. (ES8)
V64	Children can still play in the street. (GD2)
V65	There's no entertainment for young people, and they go to other towns. (GD2)
V66	The winter here is long, and there's nothing to do. (GD3)
V67	For adults, there's nothing to do, but going out to the bar. There are hardly any cultural activities; you have to go to other towns or to Valencia. (GD2)
V68	People go to other towns to have fun. GD8
**7. SERVICES**	
**Urban context**	
V69	We have more things than other cities; you can even handle all administrative procedures here. (GD1)

V70	There is a lot of doctor turnover at the hospital, which results in poor care; professionals are just passing through, and they end up leaving over time. There is only one health center for the entire city, and it's far from many neighborhoods. (GD6)
**Rural context**	
V71	You can buy practically everything in town; there's everything basic. (GD8)
V72	There are bakeries, butcher shops, a basic supermarket, and a municipal market on Thursdays where they sell clothing, fruits, and vegetables… (GD1)
V73	People go shopping by car in another town with large supermarkets where you can find everything and save money. (GD8)
V74	The respite center is the best thing that has been done in recent years. (GD8)
V75	There is no high school; you have to go to Buñol, but at least the transportation is free. (GD5)
V76	There is only a pediatrician in Buñol. (GD5)
V77	We need one more doctor at the health center. (GD2)
V78	We need high-quality bars and restaurants. Some seasons, they are all closed, and you have nowhere to go. (GD1)
**8. WORK AND LOCAL ECONOMY**	
**Urban context**	
V79	There is only work during summertime, mainly in restaurants or if you clean rooms and apartments. The pay is low, and you only work during the season and for hourly wages. What about the rest of the year? (GD4)
V80	It's harder for people over 45, especially women, to find work. (GD2)
V81	If you want to work in something other than tourism, you have to go somewhere else. (GD7)
V82	[…] During the winter, you barely get by. (GD4)
**Rural context**	
V83	There are very few companies, and therefore, very few jobs are generated. (GD1)
V84	There is no industry that promotes job creation. (GD5)
V85	The city council provides you with work for six months, and then you're unemployed. (GD2)
V86	Fewer and fewer people from outside come here, not even for vacations. (GD5)
V87	There is potential here if you revive agriculture, promote rural tourism, and establish good restaurants. (GD1)
V88	Since there are no opportunities, young people don't want to stay in town […] whether people leave depends more on jobs than housing. (GD8)
V89	Everyone works outside of town. People come here to sleep; it's a bedroom community. (ES8)
**9. HOUSING AND COMMUNITY**	
**Urban context**	
V90	People move to Las Marinas (the beach) to live because rent is cheaper in winter, but there's nothing there, not even a bus service. (GD6)
V91	Almost everything is rented out to tourists, and there are no year-round rentals; they only let you stay from October to June. (GD2)
V92	[…] They are very expensive, and there are hardly any available for the whole year; from June to September, we have to live in the campground. (GD4)

V93	We can't become independent; we have to stay at our parents' house until we can live on our own. (GD7)
**Rural context**	
V94	Rental housing is very expensive and in poor condition […] since there are no rentals, people don't come to live here; they move to Macastre. (GD2)
V95	There are many houses for sale, but they are very expensive and in poor condition. (GD1)
V96	There are many second homes owned by people who live in the city and come on weekends. (GD5)
V97	A significant portion of vacant housing is not for sale; owners prefer to let them deteriorate rather than rent them out. (GD2)
V98	There are many vacant houses, some with unfinished structures and interiors, and others are not suitable for habitation. (GD1)
**10. SOCIAL INTERACTION**	
**Urban context**	
V99	There are many associations where you can meet people. (GD5)
V100	[…] it's difficult to get to know people and make friends; the people of Denia are closed off, and it's hard to enter groups. (GD4)
V101	Everything costs money; it's easier to socialize if you have money to go for a drink, to enroll in a course, or to participate in an activity… (GD2)
V102	It's not easy for people from outside of town to meet others; there's also the language barrier, as we don't speak Valencian. (GD4)
V103	There are foreign couples who, when widowed, are left alone and disconnected. (GD1)
**Rural context**	
V104	There are many associations, *fallas*, music bands … most people belong to an association. (GD1)
V105	At the senior center, they only play bingo; you don't get to socialize with people. (ES8)
V106	There's nothing for children during the weekends, so you end up taking the car to go to the shopping center to buy things or to watch a movie. (GD3)
V107	There's nothing for young people either; there was a youth center, but it was a failure. (GD5).
**11. IDENTITY AND BELONGING**	
**Urban context**	
V108	We have the privilege of the sun and the Montgó. (GD1)
V109	It's an open city; everyone welcomes you in the end. (GD5)
V110	There are many foreigners, especially those who live on the outskirts, who don't feel like they belong here. (GD1)
V111	[...] Arab and Latin American communities feel a bit isolated; how many African or Latin *falleras* do you see in parades? (GD4)
**Rural context**	
V112	The people of Yátova are very 'yatoveros' (from Yátova). (GD5)
V113	Yatoveros are very proud of their town. (GD2)
V114	I believe it's easy to integrate; people get help to blend in, and associations do a lot […] sometimes, it's difficult for people from outside to feel like they belong here or they simply don't want to integrate. (GD2)'

V115	There is a significant detachment from the cultural heritage of the municipality, although efforts are being made to promote its knowledge and recovery. (GD1)
**12. FEELING SAFE**	
**Urban context**	
V116	This city is safe; you can go around peacefully […] occasionally, there are break-ins in some houses on Montgó, and thefts or vandalism occur. (GD6)
V117	During summertime, there are not enough police officers, and the population triples. (GD5)
V118	The nightclub and downtown pubs causes a lot of problems; there are frequent fights, vandalism, litter from drinking, people urinating in the streets… (GD6)
V119	We don't feel safe when returning home at night; we've had bad experiences. (GD2)
**Rural context**	
V120	It's a very peaceful and safe village; you don't hear about things happening, and I hardly ever lock my doors. (ES8)
V121	There are issues with vandalism, graffiti, damaging trash bins, littering, drug use … there have been robberies and damage to isolated houses in the countryside. (GD5)
V122	Not having a police presence makes you feel insecure. It's not the same as when something happens, you call them, and they have to come from another town. (GD5)
V123	Why do you need the police if nothing ever happens here? Besides, they're only five minutes away. (GD1)
**13. CARE AND MAINTENANCE**	
**Urban context**	
V124	People don't respect the garbage disposal schedules, they don't recycle properly, they don't use the containers correctly, and they litter… (GD6)
V125	The area where people gather to drink outdoors (botellón) is left in pathetic conditions with litter and broken glass in the morning; the city council cleans it up, but the following weekend, it's the same again. (GD5)
V126	[…] there is a lack of civic awareness; the administration can only do so much. (GD6)
V127	Dog feces are a problem; owners should be fined. (GD6)
**Rural context**	
V128	In general, the town is quite clean, but there are areas that could be improved. (GD2)
V129	There are some streets dirty due to dog feces and urine. (GD8)
V130	When you report a repair to the city council, they come right away. (GD5)
V131	The area where people gather to drink outdoors (botellón) is left in pathetic conditions with litter and broken glass in the morning. (GD5)
V132	The ecopark is misused, with people disposing of waste where they shouldn't. […] If recycling isn't done properly, it costs all of us money. (GD1)
**14. INFLUENCE AND SENSE OF CONTROL**	
**Urban context**	
V133	There are many ways to participate, through the app, neighborhood councilors, the city hall office, social media… (GD1)
V134	Being part of an association helps with participation. (GD5)
V135	Not many people attend meetings with neighborhood councilors. (GD6)

V136	There are no formats that encourage the participation of ordinary people. (GD5)
V137	There is no culture of participation; people don't get involved in anything. (GD6)
V138	They listen to you, but then nothing changes. (GD3)
V139	We don't complain because they don't pay attention to us; citizen participation is not real. (GD2)
V140	There are chronic problems that, for some reason, aren't being resolved. (GD6)
V141	There is a lack of information and transparency in decision-making. (GD6)
**Rural context**	
V142	Problems shouldn't be reported on Facebook. (GD1)
V143	People can go directly to the town hall or talk to the mayor on the street. (GD3)
V144	Participation is more effective through an association than individually; they pay more attention to you. (GD5)
V145	We don't feel like we are being heard; everything goes through the registry, but then nothing is done. (GD2)
V146	Young people are not paid much attention to. (GD1)
**EMERGING THEMES: TOURISM**	
**Urban context**	
V147	The good thing about summertime is that they provide bus service in this beach area, and there are more things to do than in winter, such as parties, outdoor cinema, beach activities … all for tourism. (GD6)
V148	Jobs are very precarious; there are only opportunities in the summer in the hospitality sector, and it's poorly paid. In winter, you either go on unemployment or take whatever job comes your way. (GD4)
V149	There's a strong touristification of housing; it has become much more expensive because of the high demand for vacation rentals. (GD1)
V150	During summertime, traffic is unbearable, and it's hard to find parking. Additionally, there's a lot of noise at night. (GD6)
**EMERGING THEMES: ACCESSIBILITY**	
**Urban context**	
V151	We need information about bus schedules on posters or in brochures; right now, it's only available online. […] Older people don't use the internet to check schedules; you go to the stop and wait as long as you can. (GD8)
V152	There are very few free entertainment options; everything requires payment. Whether you want to have a drink, go to a nightclub, or even play soccer, there's a cost involved. Sports and other activities are not free either. (GD7)
V153	It's hard to find out about the activities happening around here; it's not in a magazine or program that lists everything going on. (GD6)

Identifier: DG= Discussion Groups; SI=Semi-structured interview 1=XarxaSalut; 2=Women; 3= Disabled; 4=Foreigners; 5=Associations; 6=Neighborhoods; 7=Young; 8=Seniors

Supplementary Table 3. Qualitative results: Original Spanish Verbatim

**PARTICIPANTS' EXPERIENCES IN RURAL AND URBAN CONTEXTS**	
**1. WALKING OR CYCLING**	
**Urban context**	
V1	Aquí puedes ir andando a todas partes. (GD5)
V2	Hay una avenida por la que paseamos la gente mayor y no hay ni un banco. (GD8)
V3	Faltan árboles en las calles que hagan sombra y refresquen el ambiente en verano […] hay calles por las que no pasas del calor que hace. (GD8)
V4	Se necesitan aseos públicos en la carretera de Les Rotes.(GD8)
V5	Hay bancos, farolas y paneles que no deja ir bien por las aceras a las personas con sillas de ruedas, ciegas, si vas con el carrito del niño o de la compra…(GD2)
V6	En general, los carriles bici no son seguros, están llenos de baches y son muy estrechos. Muchas veces tienes que ir por la acera porque de repente se acaba el carril bici […] y a la playa tienes que ir por la carretera con los coches rozándote por el lado.(GD7)
V7	No se respetan los pasos de cebra, los coches no paran.(GD3)
V8	La zona de Les Marines es peligrosa para andar, tienes que ir por el arcén y hay mucho tráfico.(GD2)
V9	Siempre hay tráfico delante del colegio y es peligroso. (GD2)
V10	Hay sitios en los que pasas miedo con la bici, los coches no respetan las bicis.(GD5)
**Rural context**	
V11	Puedes ir andando a todas partes, esto es un pueblo pequeño. (GD2)
V12	Hay muchas calles con aceras muy estrechas, que están inclinadas, con baches… no puedes ir con la silla de ruedas o un andador, no hay rampas en ningún sitio, ni bordillos rebajados, tienes que ir por el medio de la calle. (GD3)
V13	Hay muchas cuestas y no todo el mundo puede subir al supermercado que está en la parte alta del pueblo. (GD8)
V14	Hay sitios que no tienen ni un banco para sentarse. (GD8)
V15	Si vas en bici tienes que ir por la carretera, con los coches, no hay otra forma. (GD5)
**2. PUBLIC TRANSPORT**	
**Urban context**	
V16	Hasta los hospitales están mal conectados con el autobús... si te lo puedes permitir, te coges un taxi, si no, te estás todo el día. (GD5)
V17	Es imposible ir a los pueblos de al lado como El Verger o Pedreguer, todo empieza y acaba en Denia. (GD4)
V18	Hay que llevar a los niños en coche al colegio, no hay autobús. (GD2)
V19	El centro comercial está muy mal comunicado y si, por ejemplo, trabajas allí o vas al cine y sales tarde, ya no tienes cómo volverte porque el último autobús se va pronto. (GD7)
V20	Tengo que ir andando a recoger a mis hijas del colegio porque no puedo pagar tres billetes de ida y tres de vuelta cada día.(GD2)
**Rural context**	
V21	Si no tienes coche está desconectado. (GD2)

V22	No todos los autobuses tienen rampa para silla de ruedas, tienes que llamar antes o es una lotería. (GD3)
V23	El horario del tren y el autobús no están coordinados, o llegas con mucho tiempo al tren o tienes que esperar mucho rato [...] y lo mismo para la vuelta, al final pierdes el día (GD5)
V24	No hay buena combinación para ir al hospital de Manises, te tienen que llevar en coche. (GD8)
V25	El último autobús al tren sale a las 19:20 y así es imposible trabajar a turnos o de noche en otros pueblos. (GD2)
V26	Esto es un problema de toda la comarca. (GD1)
**3. TRAFFIC AND PARKING**	
**Urban context**	
V27	La gente quiere aparcar en la puerta de casa y eso no puede ser, no hay sitio para todos. (GD6)
V28	No hay sitio para todos los coches. (GD5)
V29	Hay avenidas donde hay mucho ruido y contaminación porque pasan muchos camiones […] en verano no puedes tener las ventanas abiertas. Haría falta una ronda que los desvíe por fuera de la ciudad. (GD6)
V30	[…] se utiliza el coche para todo, hay desplazamientos que serían más rápidos andando. (GD4)
V31	Hay atascos cada día en la puerta del colegio. (GD2)
V32	En verano el tráfico está imposible. (GD5)
**Rural context**	
V33	Se puede aparcar casi en todas partes. (GD2)
V34	En verano no se cumplen las normas de circulación ni aparcamiento, pero al multiplicarse la población somos más permisivos. (GD1)
V35	Apenas hay tráfico. (GD5)
V36	La gente va en coche para ir a sitios que le cuesta lo mismo que ir andando […]hasta para llevar los niños al colegio, estando al lado. (GD8)
V37	Se está perdiendo la sana costumbre de caminar. (GD8)
**4. STREETS AND SPACES**	
**Urban context**	
V38	Depende por donde vayas hay zonas con bancos y sombra o no. (GD8)
V39	En verano hay calles por las que no puedes ir del calor que hace, deberían ponerse árboles que hagan sombra y refresquen un poco el ambiente. (GD5)
V40	Es muy agradable pasear por el centro […] el Castillo es el tesoro de la ciudad. (GD4)
V41	Debería mejorarse el alumbrado en algunas calles que tienen rincones oscuros [...]y guardar un orden en la estética de la ciudad, hay calles que cada finca es de una manera. (GD6)
**Rural context**	
V42	Es muy agradable pasear por el pueblo, esto es muy bonito, muy tranquilo. (GD2)
V43	En la plaza del pueblo hay una cuesta muy grande, estaría bien que se pusiera una barandilla. (GD8)
V44	En invierno hay algunas calles sin luz por la parte de arriba. (GD2)
V45	Faltan algunos árboles en las calles que le den otro aspecto. (GD5)
**5. NATURAL SPACES**	

**Urban context**	
V46	Los espacios naturales han mejorado mucho los últimos años, tenemos playas increíbles, la montaña aquí al lado, un parque natural, una vía verde, puedes caminar junto al mar… (GD5)
V47	Ha mejorado mucho la accesibilidad a los espacios naturales […] hasta hay una playa adaptada para personas con discapacidad. (GD1)
V48	Todos los recursos de limpieza van a las playas por el problema de las algas. (GD6)
V49	[…]faltan más parques, en el centro, en Las Marinas, solo hay cinco en total para toda la ciudad. (GD5)
**Rural context**	
V50	El ayuntamiento ha trabajado mucho para tener cuidar el entorno natural, han hecho mejoras importantes en la Cueva de Las Palomas […] hay proyectos para conservar el medioambiente y políticas orientadas a conservarlo. (GD1)
V51	La zona del Millares es un entorno a potenciar. (GD1)
V52	Tenemos una sierra preciosa a nuestro alrededor. (GD8)
V53	El parque de San Vicente está fenomenal. (GD2)
**6. PLAY AND RECREATION**	
**Urban context**	
V54	[…]faltan más actividades culturales, de teatro, conciertos… y la casa de la cultura cierra el domingo que es cuando puedes ir a cosas. (GD5)
V55	nos falta un teatro o un auditorio. (GD6)
V56	El polideportivo está en mal estado […] no puedes hacer deporte que no sea pagando. (GD5)
V57	Los colegios están cerrados los fines de semana y los niños no pueden jugar allí, donde hay canchas, campos de fútbol…(GD5)
V58	No hay nada para nosotras aparte de los bares, los pubs y la discoteca, puedes pasear calle arriba, calle abajo. (GD7)
V59	El ocio es caro, para todo tienes que pagar. (GD4)
V60	Este centro está muy bien (centro juvenil Llunàtics), es lo mejor de la comarca, aquí podemos decidir qué actividades hacer, es un sitio de encuentro, nos sentimos que aportamos y que es un poco nuestro. (GD8)
**Rural context**	
V61	Hay muchas instalaciones deportivas, tenemos gimnasio, frontón, canchas de fútbol y basket… hay clases de pilates, zumba, karate… y hay grupos de gente que sale a caminar. (GD1)
V62	En el centro de jubilados pagas una cuota y solo juegas al bingo, no hay nada más… si todos los días hubiera bingo, todos los días estaría lleno.(EE8)
V63	No hay nada para la gente mayor y acabas todo el día cara a la televisión.(EE8)
V64	Los niños todavía pueden seguir jugando en la calle.(GD2)
V65	No hay oferta de ocio para la gente joven y se van a otros pueblos.(GD2)
V66	El invierno aquí es largo y no hay nada que hacer.(GD3)
V67	Para los adultos no hay nada, ir al bar. Apenas hay cosas culturales, hay que ir a otros pueblos o a Valencia.(GD2)
V68	La gente se marcha a otros pueblos a divertirse.(GD8)
**7. SERVICES**	

**Urban context**	
V69	Tenemos más cosas que otras ciudades, incluso puedes hacer todas las gestiones aquí (administrativas). (GD1
V70	Hay mucha rotación de médicos en el hospital y eso deriva en mala atención, los profesionales están de paso, se acaban yendo al tiempo […] solo hay un centro de salud para toda la ciudad y queda lejos de muchos barrios. (GD6)
**Rural context**	
V71	Se puede comprar prácticamente de todo en el pueblo, hay de todo lo básico. (GD8)
V72	Hay panaderías, carnicerías, supermercado básico y mercado municipal los jueves, donde traen ropa, fruta, verdura… (GD1)
V73	La gente se va a hacer la compra en coche a otro pueblo que tienen supermercados grandes donde hay de todo y se ahorran dinero. (GD8)
V74	El centro de respiro es lo mejor que se ha hecho los últimos años. (GD8)
V75	No hay instituto, hay que ir a Buñol pero al menos el transporte es gratuito. (GD5)
V76	Solo hay pediatra en Buñol. (GD5)
V77	Falta un médico más en el centro de salud. (GD2)
V78	Hacen falta bares y restaurantes de calidad. Hay temporadas que están todos cerrados y no tienes donde ir. (GD1)
**8. WORK AND LOCAL ECONOMY**	
**Urban context**	
V79	Solo hay trabajo en verano y en restaurantes o limpiando habitaciones y apartamentos, se paga poco y solo trabajas en temporada y por horas, ¿y el resto del año qué? (GD4)
V80	Los mayores de 45 años, sobre todo a las mujeres, nos es más difícil encontrar trabajo. (GD2)
V81	Si quieres trabajar en otra cosa que no sea para el turismo, te tienes que ir a otro sitio. (GD7)
V82	[…]en invierno malvives. (GD4)
**Rural context**	
V83	Hay muy pocas empresas y, por tanto, se genera poco trabajo. (GD1)
V84	No hay industria que favorezca la creación de empleo. (GD5)
V85	El ayuntamiento te da trabajo 6 meses y después a la calle. (GD2)
V86	Cada vez viene menos gente de fuera, ni siquiera de vacaciones. (GD5)
V87	Aquí hay potencial si recuperas la agricultura, con el turismo rural y poniendo buenos restaurantes. (GD1)
V88	Como no hay oportunidades, la gente joven no quiere quedarse en el pueblo[…]que la gente se vaya depende más del trabajo que de la vivienda. (GD8)
V89	Todo el mundo trabaja fuera del pueblo. La gente viene a dormir, esto es un pueblo dormitorio. (EE8)
**9. HOUSING AND COMMUNITY**	
**Urban context**	
V90	La gente se va a Las Marinas (la playa) a vivir porque el alquiler es más barato en invierno, pero allí no hay de nada, ni siquiera llega el autobús. (GD6)
V91	Casi todo se alquila al turismo y no hay alquileres para el año entero, solo te dejan quedarte de octubre a junio. (GD2)
V92	[…]son carísimos y apenas hay para todo el año, de junio a septiembre nos

	tenemos que ir a vivir al camping. (GD4)
V93	Nno nos podemos independizar, nos tenemos que quedar en casa de nuestros padres hasta que nos podamos marchar de aquí. (GD7)
**Rural context**	
V94	Las viviendas de alquiler son muy caras y están en condiciones precarias[…]como no hay alquiler, la gente no viene a vivir aquí, se marchan a Macastre. (GD2)
V95	Hay muchas casas en venta, pero muy caras y en malas condiciones. (GD1)
V96	Hay mucha segunda vivienda de personas que viven en la ciudad y vienen los fines de semana. (GD5)
V97	Gran parte de la vivienda vacía no se quiere vender, los dueños prefieren que se caigan antes que alquilarlas. (GD2)
V98	Hay muchas casas vacías, algunas con la estructura y el interior por terminar y otras sin acondicionar. (GD1)
**10. SOCIAL INTERACTION**	
**Urban context**	
V99	Hay muchas asociaciones donde puedes conocer a gente. (GD5)
V100	[…]cuesta conocer a gente y hacer amigos, la gente de Denia es cerrada y cuesta entrar en grupos. (GD4)
V101	Todo cuesta dinero, es más fácil relacionarte si tienes dinero para tomarte algo, para apuntarte a un curso, a alguna actividad… (GD2)
V102	No es fácil para la gente que viene de fuera conocer a otra gente, además está la barrera del idioma, no hablamos valenciano. (GD4)
V103	Hay parejas de extranjeros que al enviudar se quedan solos y desconectados. (GD1)
**Rural context**	
V104	Hay muchas asociaciones, fallas, bandas de música… la mayoría de la gente pertenece a alguna asociación. (GD1)
V105	En el centro de jubilados solo se juega al bingo, no te relacionas con gente. (EE8)
V106	El fin de semana no hay nada para los niños, así que te acabas cogiendo el coche para ir al centro comercial a comprar o al cine. (GD3)
V107	Para la gente joven tampoco hay nada, hubo un centro juvenil, pero fue un fracaso. (GD5).
**11. IDENTITY AND BELONGIN**	
**Urban context**	
V108	Tenemos el privilegio del sol y del Montgó. (GD1)
V109	Es una ciudad abierta, todo el mundo te acoge al final. (GD5)
V110	Hay muchos extranjeros, sobre todo los que viven a las afueras, que no se sienten de aquí. (GD1)
V111	[…] las comunidades árabes y latinoamericanas nos vemos un poco aisladas, ¿a cuántos falleras africanas o latinas has visto en los desfiles? (GD4)
**Rural context**	
V112	La gente de Yátova es muy yatovera. (GD5)
V113	Los yatoveros estamos muy orgullosos de nuestro pueblo. (GD2)
V114	Creo que es fácil integrarse, se ayuda a la gente a que se integre, las asociaciones hacen mucho […] a veces a la gente de fuera le cuesta sentirse de aquí o directamente no quiere integrarse. (GD2)

V115	Hay gran desapego hacia al patrimonio cultural del municipio, aunque se está fomentando su conocimiento y recuperación. (GD1)
**12. FEELING SAFE**	
**Urban context**	
V116	Esta ciudad es segura, puedes ir tranquilamente […] de vez en cuando entran en alguna casa del Montgó y roban o rompen cosas. (GD6)
V117	En verano no hay más policías y se triplica la población. (GD5)
V118	La discoteca del centro genera muchos problemas, hay peleas cada dos por tres, vandalismo, suciedad por el botellón, orinan en la calles… (GD6)
V119	No nos sentimos seguras al volver a casa por la noche, hemos tenido malas experiencias. (GD2)
**Rural context**	
V120	Es un pueblo muy tranquilo y seguro, no se oye que pasen cosas, yo casi nunca echo la llave. (EE8)
V121	Hay problemas de vandalismo, pintadas, rompen alguna papelera, ensucian, toman drogas… […] ha habido robos y destrozos en las casas aisladas en el campo. (GD5)
V122	No tener policía te da inseguridad. No es lo mismo que te pase algo, la llames, y tenga que venir de otro pueblo. (GD5)
V123	¿Para qué quieres policía si nunca pasa nada aquí?, además están a 5 minutos. (GD1)
**13. CARE AND MAINTENANCE**	
**Urban context**	
V124	La gente no respeta los horarios para tirar la basura, no hacen bien el reciclaje, no utilizan bien los contenedores, tiran la basura por ahí… (GD6)
V125	La zona de botellón amanece hecha una pena de suciedad, cristales rotos… lo limpia el ayuntamiento, pero al siguiente fin de semana otra vez igual. (GD5)
V126	[…]falta concienciación ciudadana, la administración llega hasta donde puede. (GD6)
V127	las cacas de los perros son un problema, se debería multar a los dueños. (GD6)
**Rural context**	
V128	En general, el pueblo está bastante limpio, pero hay zonas para mejorar. (GD2)
V129	Hay algunas calles sucias por los excrementos y orines de los perros. (GD8)
V130	Cuando avisas al ayuntamiento sobre alguna reparación vienen en seguida. (GD5)
V131	La zona de botellón amanece hecha una pena de suciedad, cristales rotos… (GD5)
V132	Se hace mal uso del ecoparque tirando residuos donde no se debe […]si no se hace bien reciclaje, nos cuesta dinero a todos. (GD1)
**14. INFLUENCE AND SENSE OF CONTROL**	
**Urban context**	
V133	Hay muchas formas de participar, por la APP, los concejales de barrio, la oficina del ayuntamiento, las redes sociales… (GD1)
V134	Pertenecer a una asociación ayuda a participar. (GD5)
V135	No acude mucha gente a las reuniones con los concejales de barrio. (GD6)
V136	No hay formatos que fomenten la participación de la gente de a pie. (GD5)
V137	No hay cultura de participación, la gente no se involucra en nada. (GD6)
V138	Te escuchan, pero luego no cambia nada. (GD3)
V139	No nos quejamos porque no nos hacen caso, la participación ciudadana no es real.

	(GD2)
V140	Hay problemas cronificados que no sabemos por qué no se solucionan. (GD6)
V141	Falta información y transparencia en la toma de decisiones. (GD6)
**Rural context**	
V142	Los problemas no deberían denunciarse en Facebook. (GD1)
V143	La gente puede ir directamente al ayuntamiento o hablar con el alcalde en la calle. (GD3)
V144	Se participa más desde una asociación que de forma individual, te hacen más caso. (GD5)
V145	No nos sentimos escuchadas, todo pasa por registro, pero luego no se hace nada. (GD2)
V146	A los jóvenes no se le hace mucho caso. (GD1)
**EMERGING THEMES: TOURISM**	
**Urban context**	
V147	Lo bueno que tiene el verano es que ponen autobús en esta zona (de playa) y hay más cosas que hacer que en invierno, hay fiestas, cine de verano, actividades en la playa… todo por el turismo. (GD6)
V148	El trabajo es muy precario, solo hay en verano en la hostelería y mal pagado, y en invierno te vas a al paro o a lo que salga. (GD4)
V149	Hay una turistificación de la vivienda, se ha encarecido muchísimo porque hay mucho alquiler turístico. (GD1)
V150	En verano el tráfico es imposible y no puedes aparcar, además de que hay mucho ruido por las noches. (GD6)
**EMERGING THEMES: ACCESSIBILITY**	
**Urban context**	
V151	Hace falta información de los horarios del autobús en carteles en las paredes o en folletos, ahora solo está en internet […] las personas mayores no entramos en internet a mirar horarios, vas a la parada y esperas lo que puedas. (GD8)
V152	Hay muy poquitas opciones de entretenimiento que sean gratis, para todo hay que pagar, o tienes que ir a tomarte algo, o la discoteca, ni jugar al fútbol es gratis, ni deporte ni nada. (GD7)
V153	No te enteras de las actividades que se hacen por ahí, no está en una revista o un programa donde ponga todo lo que se hace. (GD6)

Identificador: GD= Grupo de discusión; EE=Entrevista estructurada 1= XarxaSalut; 2= Mujeres en situación vulnerable; 3= Personas con discapacidad; 4= Personas extranjeras; 5= Asociaciones; 6= Barrios; 7= Jóvenes; 8= Mayores
